# In-depth phenolic characterization of iron gall inks by deconstructing representative Iberian recipes

**DOI:** 10.1038/s41598-021-87969-3

**Published:** 2021-04-23

**Authors:** Natércia Teixeira, Paula Nabais, Victor de Freitas, João A. Lopes, Maria J. Melo

**Affiliations:** 1 LAQV/REQUIMTE, Department of Chemistry and Biochemistry, Faculty of Sciences, Universidade do Porto, Rua do Campo Alegre, s/n, 4169-007 Porto, Portugal; 2 LAQV/REQUIMTE, Department of Conservation and Restoration, Nova School of Science and Technology, 2829-516 Monte da Caparica, Portugal; 3 iMed.ULisboa-Research Institute for Medicines, Faculty of Pharmacy, University of Lisbon, Av. Prof. Gama Pinto, 1649-003 Lisbon, Portugal

**Keywords:** Chemistry, Chemistry publishing, Organic chemistry

## Abstract

Iron-gall ink is one of the most important inks in the history of western civilization. The deep black colour results from Fe^3+^ complexes with phenolic compounds available in gall extracts. Unfortunately, it induces the degradation of both ink and support over time. Furthermore, our knowledge of these complex molecular structures is limited. This work aims to overcome this gap, revealing essential information about the complex structures of these pigments and dyes that will create a breakthrough in the next generation of conservation treatments. It presents the first in-depth phenolic identification and quantification of extracts and inks, prepared with and without gum arabic (an essential additive in medieval recipes). Five representative Iberian recipes were selected and prepared. Their phenolic profile was analysed by HPLC–DAD and HPLC–ESI–MS, which revealed that the phenolic compounds present in higher concentration, in the gall extracts, are pentagalloylglucose and hexagalloylglucose (0.15 ± 0.01–32 ± 3 mg/mL), except for one recipe, in which gallic acid is the main phenolic. The influence of the ingredients is also discussed by deconstructing the recipes: extracts of additives as pomegranate peel and solvents used in the extraction of the galls (vinegar and white wine) were characterized.

## Introduction

Iron gall inks were commonly used for writing or drawing until the beginning of the twentieth century. Hand-written documents, manuscripts, music scores and painting sketches form a fundamental part of our cultural heritage and were created using iron gall inks. These inks were chosen to substitute, in part, carbon black inks that were more prone to detachment from its physical support^1^. They could penetrate and bind to paper or parchment and so becoming more permanent. After hundreds of years later, this becomes a disadvantage as these inks began to degrade the support^2–4^. The cause behind this phenomenon lies on its colorant. The black colour characteristic of iron gall inks is given by the formation of Fe^3+^-polyphenol dark complexes. The large variety of different recipes and the compositional diversity of the used natural materials result in a diversity of degradation mechanisms leading to changes to a brownish colour over time, resulting in iron gall ink corrosion and paper degradation that implies the loss of the written information^5^.

Conservators and material scientists agree that acid-catalysed hydrolysis and metal-catalysed oxidation are the major chemical processes that are responsible for the loss of mechanical strength of the paper support^5,6^. Two principal causes are pointed out to be responsible for this paper degradation: the high acidity of some inks that leads to hydrolytic scission of the polymer chain (for this reason, in this work, the pH values of the final inks and extracts are reported); and the presence of soluble and mobile iron ions (Fe^2+^) that may act as catalysts for oxidative scission of cellulose^5^. Spectroscopic studies using several inks made with tannins indicate that different plant sources for galls lead to different spectral features of inks^7^. Consequently, different ink–substrate interactions and degradation pathways can be observed^7–9^.

Sustainable preservation strategies are crucial to preserve these inks, and a great research effort was made over the last years to investigate chemical treatments capable of delaying paper degradation induced by iron gall inks^10^. From a heritage science perspective, the identification of the iron gall ink composition is important in order to understand the mechanisms of degradation^11^. In fact, our knowledge of the molecular structures of the chemical compounds present in medieval inks is very limited. This gap prevents us from devising informed strategies for preserving the world written heritage.

**Figure 1:**
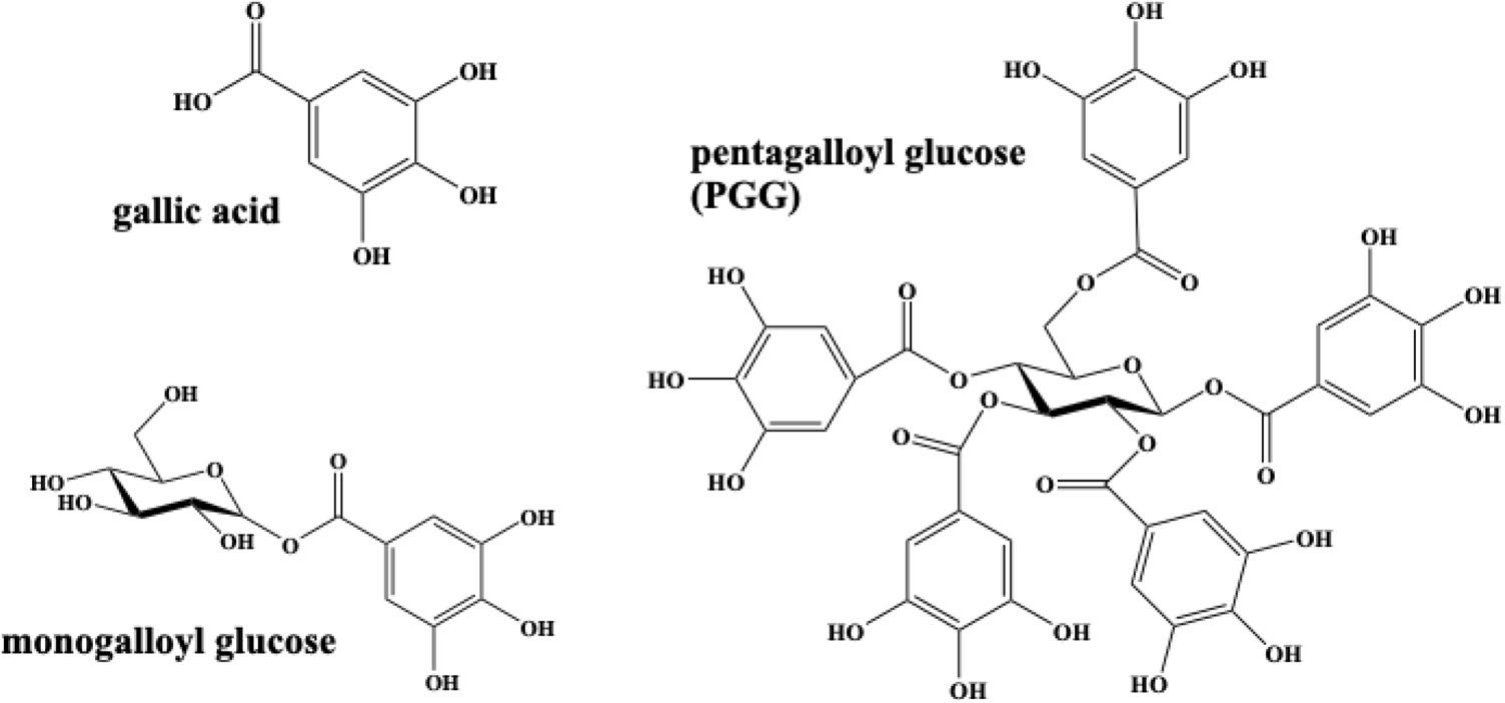
Molecular structures of gallic acid, monogalloyl glucose and pentagalloyl glucose (PGG).

The understanding of colour stability relies on the reproduction of these ancient and complex systems. For this reason, we started this research with the reconstruction of medieval writing inks based on technical sources that allow us to produce them in the laboratory with as much historical accuracy as possible^12^. In medieval written sources^12,13^, iron gall ink recipes contain the three basic ingredients depicted in Supplementary Scheme S1. Plant extracts such as *Quercus infectoria* were mixed with iron salts (e.g., FeSO_4_) to produce a dark iron–polyphenol complex, to which gum arabic was usually added to keep the pigment in suspension and to make the ink more suitable for writing^1,10^. Different additives, such as other metal ions, pigments, solvents as well as different extraction conditions are commonly described in these recipes^12,14^. The results of this research, in medieval inks, show that polygalloyl esters of glucose are the main phenolic compounds available in gall extracts to complex Fe^3+^, and that free gallic acid is a minor component in the extracts and inks^12^ (Fig. 1). So, before continuing using models based on iron-gallates for new conservation and preservation strategies, a complete study of the chemical composition of the gall extracts and inks is needed. This knowledge will allow great progress in the study of iron coordination, which dictate the stability of the inks, also integrating results from other fields of research, whenever possible. It is already known that the catechol or pyrogalloyl ring with 2 or 3 hydroxyl groups respectively, provide binding sites for the chelation of metal ions^15^ (Fig. 1). It is known that these phenolic ligands stabilize by complexation Fe^3+^ ions over Fe^2+^. The oxidation of Fe^2+^ is well documented in food and health science research, and Dangles et al. have already proven that the higher metal-binding stability constants for Fe^3+^ are the reason for this oxidation to occur. Meanwhile, Fe^3+^ reduction may form quinone or semiquinone species preventing ester hydrolysis and the release of free gallic acid^16,17^.

Therefore, the major goal of this work was to identify and determine the quantification of individual phenolic compounds found on gall extracts and respective inks following five medieval recipes, which were selected based upon research into Iberian written sources of medieval techniques^12^. The analysis was conducted by HPLC–ESI–MS chromatography for the compound identification and HPLC–DAD for the quantification of those compounds. The variation of the concentration of these compounds in the gall extracts, ink with and without gum arabic was also calculated and compared.

## Results and discussion

All recipes were reproduced following Table 1 (“Materials and methods”), and the detailed recipes are already published elsewhere^12^. The diversity in the typology of the recipes, will allow to assess the influence of the extraction system, the effect of other added salts, such as copper sulphate, as well as the effect of a diversity of additives that are called for in certain recipes such as alum, indigo, pomegranate peel. The extraction systems selected cover a great diversity of solvents (water, water + vinegar, water + wine, wine), temperature ranges: from room temperature to boiling, as well as extraction times: from "boil and reduce to half" up to 3, 6 or 9 days extracting at room temperature.

**Table 1:** Composition and experimental conditions used to prepare the five iron-gall inks from the Iberian treatises (adapted from Ref.^12^).

Manuscript	Galls	Solvent	FeSO_4_	Galls: FeSO_4_: Gum	Other	Extraction	Filtration
Braga	*Quebrantar* Ground	Water:Vinegar 2:1 (15.90 mL/1 g galls)	*Azeche*	1:4:0.5	–	Boil, reduce 2×	No
Montpellier	*Romper* Crush	Water (34.96 mL/1 g galls)	*Acije*	1:0.6:0.6	–	3 days Boil, reduce 4×	Yes
Cordoba	*Quebrantar* Ground	Water (26.07 mL/1 g galls)	*Asiche*	1:1:0.5	Pomegranate peel (0.5)	8 days RT Boil	Yes
Guadalupe	*Partidas* Crushed	Water:White wine 1:0.25 (8.93 mL/1 g galls)	*Azige*	1:0.6:0.3	CuSO_4_ (0.3)	6 days Heat	Yes
Madrid	*Quebrantar* Ground	White Wine (22.62 mL/1 g galls)	*Caparrosa*	1:1:1	Alum (0.08) + Indigo (0.08) + Brown Sugar (0.08)	9 days RT	Yes

Original text recipes and transcriptions are available in Supplementary information. All ratios were calculated using the compounds' mass.

*RT* room temperature.

The term “extract” will be used referring to the final extract according to the recipe demands. When the recipe demands the heating of that extract, the extract prior heating was also analysed for comparison with the heated final extract.

### Phenolic compound analysis and quantification

**Figure 2:**
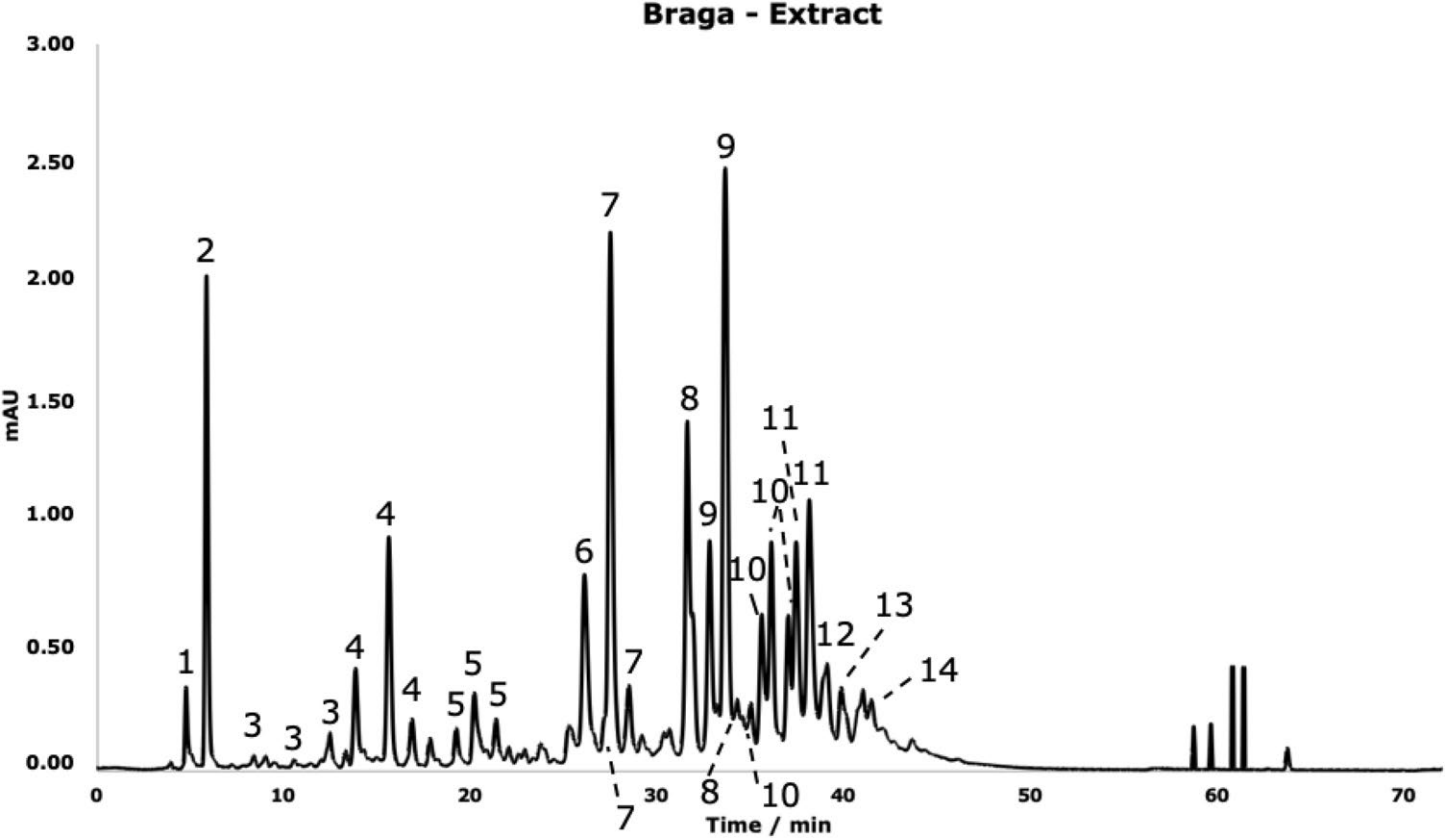
HPLC–ESI–MS chromatogram for the Braga extract, obtained using the gallotannin method (for more details, please see “Materials and methods”).

All extracts and inks were analysed by HPLC–ESI–MS to allow the identification of different phenolic compounds. The phenolics identified in the Braga extract are indicated in Fig. 2 and Table 2 in which are represented the results for all the analysed extracts.

**Table 2:** HPLC–ESI–MS identification of the phenolic compounds present in the Braga extract.

Compound		R_t_/min	[M−H]^−^ (*m/z*)	[M−2H]^2−^ (*m/2z*)	MS^2^ (*m/z*)	MS^3^ (*m/z*)
1	Gallic acid glucoside	4.77	331		271; 241; 169	211
2	Gallic acid	5.47	169		125	
3	Digalloylglucose	7.27	483		331; 313; 169; 271; 193; 211	169; 271; 211;193; 125
		10.28	483			
		11.03	483			
4	Digallic acid	12.11	321		169	125
		13.93	321			
		14.56	321			
5	Trigalloylglucose	17.15	635		483; 465	271; 331; 313; 211; 169
		17.92	635			
		19.41	635			
6	Trigallic acid	23.88	473		321	169
7	Tetragalloylglucose	24.49	787		617; 635; 573; 421; 465	573; 465; 447; 529; 313
		25.27	787			
		26.24	787			
8	Pentagalloylglucose	29.53		469	393; 169	317; 169
9		30.64	939		787	617; 635; 573; 465; 529
		31.51	939			
8		32.52		469	393; 169	317; 169
10	Hexagalloylglucose	32.93		545	469	393; 317; 169
		33.64		545	469	393; 317; 169
		34.15		545	469	393; 317; 169
		34.87		545	469	393; 317; 169
11		35.36	1091		787; 939	617; 635; 573; 465
		36.23	1091			
12	Heptagalloylglucose	37.96		621	545	469
13		38.95	1243		939; 1091	
14	Diethyl gallate	39.45	349		197	169; 125

HPLC–ESI–MS analysis allowed the tentative identification of several gallotannins and gallic acid derivatives. By observing the pseudomolecular ion fragments of the compounds *m/z* [M]^−^ 169 and *m/z* [M]^−^ 331 it is possible to identify them as gallic acid (**2**) and gallic acid glucoside (**1**) respectively, since MS^2^ spectra show pseudomolecular ions with *m/z* 125 and *m/z* 169 (a − 162 m*/z* loss corresponding to the cleavage of a glucose moiety)^18^.

Compound **4** with *m/z* 321 correspond to digallic acid since the MS^2^ and MS^3^ spectra revealed the presence of pseudomolecular ions with *m/z* 169 and *m/z* 125 corresponding to gallic acid^19^. The same principle was followed on the identification of compound **6** as trigallic acid.

It was possible to identify 6 types of gallotannins in this extract. [M−H]^−^ at *m/z* 483 has been assigned to digalloylglucose (**3**) as the MS^2^ fragmentation pattern reveals pseudomolecular ions with *m/z* 313 corresponding to [M−170], the loss of a gallate residue, and *m/z* 331 corresponding to [M−152], the loss of a galloyl residue. The pseudomolecular ion with *m/z* 635 is compatible with the structure of trigalloylglucose (**5**) for the MS^2^ spectra shows pseudomolecular ions with *m/z* 483 and 465 corresponding to the loss of a galloyl (originating a digalloylglucose pseudomolecular ion) and a gallate unit^20^. The same principle was applied to the identification of pseudomolecular ions *m/z* 787, 939, 1091 and 1243 as tetragalloylglucose (**7**), PGG (**9**), hexagalloylglucose (**11**) and heptagalloylglucose (**13**) respectively.

Compounds **8**, **10** and **12** were identified as PGG, hexagalloylglucose and heptagalloylglucose isomers due to double charged pseudomolecular ions [M−2H]^2−^ at: *m/2z* 469 (half value + 1 of [M−H]^−^ *m/z* PGG 939), with a MS^2^ spectra that shows pseudomolecular ions with *m/z* 393 and *m/z* 169 (gallic acid residue); *m/2z* 545, with MS^2^ pseudomolecular ion *m/z* 469 and MS^3^ spectra pseudomolecular ion *m/z* 393 and *m/z* 169; and *m/2z* 621, with MS^2^ pseudomolecular ion *m/z* 545 and MS^3^ spectra pseudomolecular ion *m/z* 469. This behaviour on negative mode MS analysis is typical for gallotannins and it has already been described elsewhere^21^. It can be explained due to isotopic distribution were the peaks spaced by 1 a.m.u. (atomic mass unit) correspond to the difference between naturally occurring ^12^C and ^13^C isotopes, and peaks spaced by less are assigned to multicharged pseudomolecular ions (Supplementary Fig. S1).

After the identification of the major phenols present in extracts and all the inks, it is important to quantify them to allow a full comparison between the different recipes tested.

**Figure 3:**
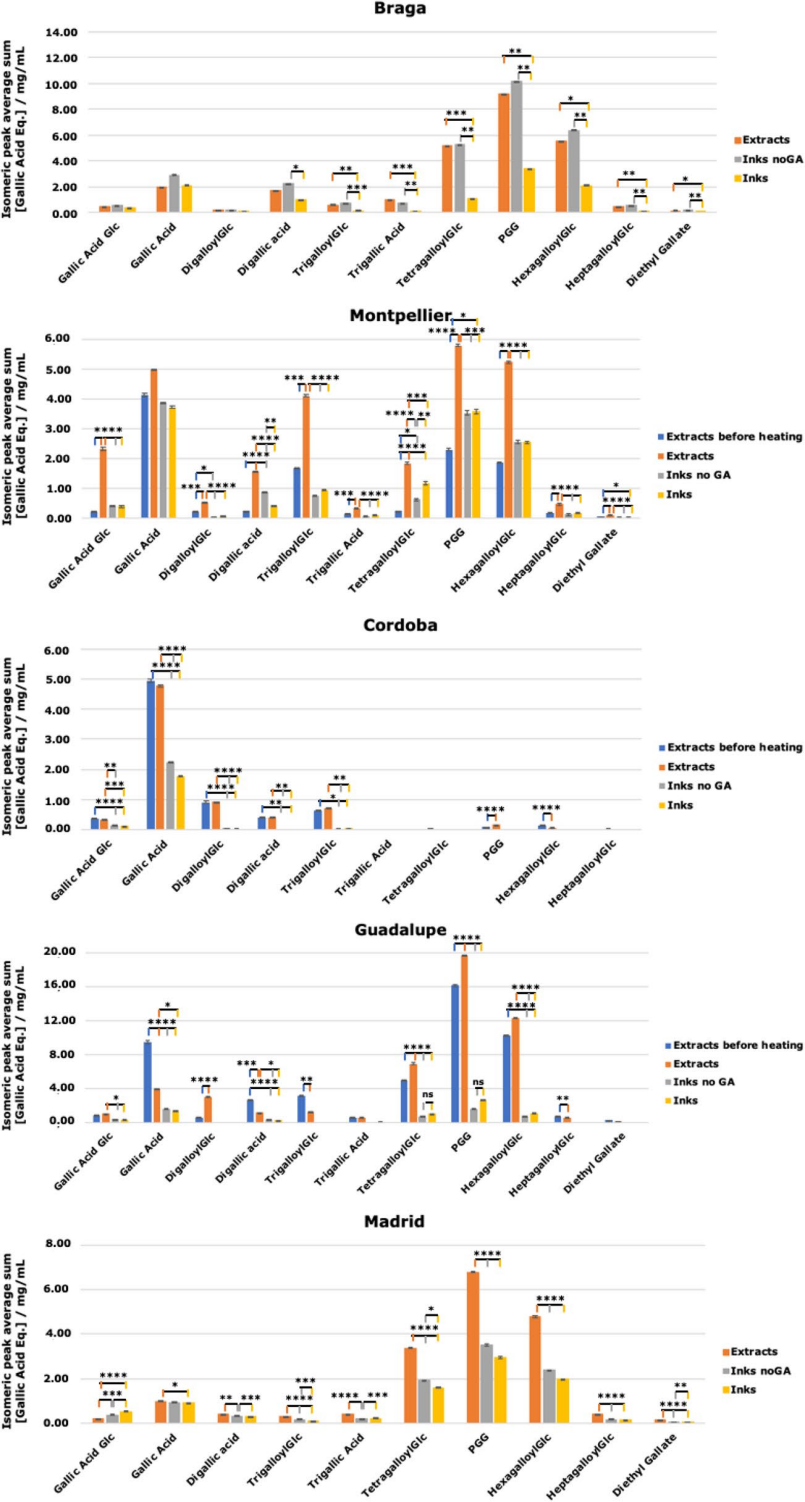
Concentration of the phenolic compounds (expressed in mg/mL of equivalents of gallic acid) identified in the extracts and inks (with and without gum arabic) for all the recipes. Statistical significance P < 0.05.

Figure 3 represents the comparison of the concentration of all the phenolic compounds (expressed in mg/mL of equivalents of gallic acid) identified in all extracts and inks (with and without gum arabic) for all recipes. To simplify, the concentration of isomeric compounds was summed. Table 3 represents the concentration of gallic acid and phenolic compounds present in higher concentration (PGG and HGG), as well as the sum of all phenolic compounds and ratio gallic acid/sum of phenolic compounds found in all extracts and inks following the 5 medieval recipes. Supplementary Tables S1–S5 represent the concentration of all individual phenols found in all extracts and inks for the 5 recipes.

**Table 3:** Concentration of gallic acid and sum of PGG and hexagalloylglucose (HGG) (expressed in mg/mL of equivalents of gallic acid), sum of all phenolic compounds and ratios: gallic acid/sum of phenolic compounds and PGG + HGG/sum of phenolic compounds found in the extracts and inks following the 5 medieval recipes.

Recipe	[Gallic acid]	[PGG + HGG]	Σ Phenolic compounds	% ([Gallic Acid]/Σ Phenolic compounds)	% ([PGG + HGG]/Σ Phenolic compounds)
**Extracts**					
Braga	1.9 ± 0.6	15 ± 2	26 ± 3	7.7 ± 0.9	56 ± 2
Montpellier	5 ± 1	11 ± 1	27 ± 2	18 ± 2	40 ± 6
Cordoba	5 ± 1	0.15 ± 0.01	7 ± 4	65 ± 3	2.1 ± 0.8
Guadalupe	4 ± 1	32 ± 3	51 ± 4	8 ± 1	62 ± 3
Madrid	1.00 ± 0.04	11.5 ± 0.5	17.8 ± 0.6	5.6 ± 0.3	65 ± 1
**Inks without Gum Arabic**					
Braga	2.9 ± 0.5	17 ± 1	30 ± 3	10 ± 1	55 ± 2
Montpellier	4 ± 1	6 ± 1	13 ± 2	30 ± 4	47 ± 4
Cordoba	2.2 ± 0.2	0.03 ± 0.01	2.5 ± 0.2	88 ± 1	1.0 ± 0.4
Guadalupe	1.7 ± 0.1	2 ± 1	6 ± 1	29 ± 3	38 ± 7
Madrid	0.94 ± 0.04	5.9 ± 0.9	10 ± 1	9 ± 1	58 ± 3
**Inks with Gum Arabic**					
Braga	2.1 ± 0.6	6 ± 1	11 ± 3	20 ± 3	52 ± 2
Montpellier	3.7 ± 0.4	6 ± 1	13 ± 2	28 ± 3	47 ± 3
Cordoba	1.8 ± 0.1	0.04 ± 0.02	2.0 ± 0.2	87 ± 2	2 ± 1
Guadalupe	1.4 ± 0.1	4 ± 1	7 ± 2	20 ± 2	53 ± 4
Madrid	0.9 ± 0.1	4.9 ± 0.5	8.9 ± 0.8	10 ± 1	56 ± 1

One important aspect to notice is that, by analysing the inks by HPLC under the conditions mentioned in “Materials and methods”, only the free phenolic compounds are analysed and not the phenol–Fe complexes, since no additional peaks were detected using these HPLC methods. To study the Fe-complexation capability, a simple test using standard gallic acid and PGG as the extract was performed: the galls:FeSO_4_:gum arabic proportions described for each recipe were maintained, but no heat was applied. The inks were analysed by HPLC (using the gallotannin method) and the results are shown in Supplementary Fig. S2. PGG led to a much higher concentration decreasing than gallic acid, meaning that the complex formed with PGG seems to be more stable than the complex formed with gallic acid. Other aspects such as complex formation kinetic, complex stability or solubility may be interfering with this test. Further studies will be conducted on the stability of the phenol–Fe complexes under the conditions of the HPLC method used.

The quantity of phenolic compounds, in the gall extracts, range from 7.5 (Cordoba) to 51 (Guadalupe), expressed in mg/mL of equivalents of gallic acid (Table 3). Guadalupe is thus the recipe with the highest concentration of phenolic compounds in the extract, after heating; Braga, Montpellier and Madrid display *ca* half this concentration. When iron sulfate is added, and the black chromophore is formed, these amounts decrease, with the exception of the Braga recipe where no variation is observed within the experimental error (Table 3). The highest decrease is observed for the recipe that extracted the highest quantity of polyphenols, Guadalupe, where a decrease from 51 to 6 mg/mL is observed. It is possible that the high concentration of phenolic compounds extracted allowed the formation of insoluble networks in much higher concentration than for the other inks.

The addition of gum arabic did not induce further variations in the concentration of phenolic compounds, except for the Braga recipe (which is the recipe that uses the highest amount of FeSO_4_).

The inks prepared following the recipes Braga and Montpellier present higher concentrations of digallic acid. Meanwhile, the extracts Cordoba and Guadalupe although showing high amounts of this compound, it greatly decreases when FeSO_4_ is added. Madrid, Braga and Guadalupe are the recipes that show the higher concentrations of tetragalloylglucose. Guadalupe and Cordoba are the recipes with the highest concentration of gallic acid in the extracts before and after heating.

As indicated in the literature^22^ alcohol from wine and spirits helps to increase the phenolic content in gall extracts. Interestingly, in the recipe where 100% white wine is used (Madrid), the percentage of phenolics extracted is much lower comparing to the recipe Guadalupe that uses only 25% of white wine as extract solvent.

Both Montpellier and Cordoba extracts are prepared in water. However, the Cordoba extract, which also displays the lowest concentration of phenolic compounds is composed mainly by gallic acid, contrary to what is observed in the Montpellier extract (Table 3). To better understand the efficacy of the extraction process and the effects of pomegranate peel in the evolution of the phenolic profile, two new Montpellier extracts were prepared, using the same extraction time of Cordoba, of 8 days at room temperature, with and without the addition of pomegranate peel and boiled until the volume was reduced to 1/4. The results are shown in Supplementary Fig. S3.

By comparing Fig. 3 and Supplementary Fig. S3, it is clear that the concentration of gallic acid and other detected compounds in the Montpellier extract is much higher in the 8-day extract than the concentrations of the 3-day extract. Also, the extract with no addition of pomegranate peel shows a higher concentration of gallic acid than the extract with it, and a lower concentration of digallic acid (almost half the concentration). These results show that the extraction time influences the final phenolic profile and that the addition of pomegranate also plays a role in it. In Supplementary Fig. S4 is possible to observe that the extract without the addition of pomegranate peel is much darker. These effects will be further explored as future work.

### Other added ingredients

Four of the five recipes studied use other ingredients besides the already mentioned three base-ingredients. These extra-ingredients were analysed focusing on the content of compounds with catechol or pyrogallol moieties in their chemical structure.

Freeze-dried pomegranate peel was added to the recipe Cordoba. The HPLC–ESI–MS analysis of the extract Cordoba and the quantification of the compounds only originated from the pomegranate peel is shown in Table 4a.

**Table 4:** HPLC–ESI–MS characterization of organic acids and phenolic compounds bearing a catechol or a pyrogallol moiety present in the extracts of other added ingredients to the inks.

Compound	R_t_/min	[M−H]^−^	MS^2^ (*m/z*)	[gallic acid] eq./mg/mL		
**(a) Pomegranate Peel**						
Punicalin	5.33	781	721; 601; 299	0.084 ± 0.003		
Punicalagin	11.02	1083	781; 601; 301	0.104 ± 0.009		
Ellagic acid	27.77	301	257; 229; 185	0.0019 ± 0.0001		

Compound	R_t_/min	[M−H]^−^ (*m/z*)	[2M−H]^−^ (*m/z*)	MS^2^ (*m/z*)	MS^3^ (*m/z*)	[gallic acid] eq./mg/mL
**(b) White Wine**						
Tartaric acid	6.24	149		87		
Caffeic acid	9.00	179		135		3.0 ± 0.2
Malic acid	10.05	267		133	89; 71; 87	
	10.95	267		133	75; 89; 115	
Cinnamic acid	12.00	147		129; 85; 87; 101	85	
Gallic acid	17.23		339	169	125	2.2 ± 0.4
Methyl coumarate	18.56		355	177	103; 131; 157; 85	
Diethyl coutaric acid	26.27		323	161	143; 115; 71	
Methyl cinnamate	29.38	161		143; 115; 71; 87	71; 98	
Caftaric acid	35.50		623	311;179	149; 179; 135	3.0 ± 0.4
Ethyl cinnamate	40.18	175		129; 85; 157	85; 101	
	41.31	175		129; 85; 157	85; 101	
Coutaric acid	44.92	295		163; 149	119	
	47.12	295		163; 149	119	
Methyl coumarate	56.21	177		103; 131		
Ethyl gallate	67.84	197		169; 125		1.4 ± 0.2
Quercetin Glucuronide	87.88	477		301	179; 151; 273; 257	0.31 ± 0.02

Compound	R_t_/min	[M−H]^−^ (*m/z*)	[2M−H]^−^ (*m/z*)	MS^2^ (*m/z*)	MS^3^ (*m/z*)	[gallic acid] eq./mg/mL
**(c) White wine vinegar**						
Caffeic acid	9.01	179		135		0.81 ± 0.07
Sinapic acid	9.53	223		133		
Malic acid	9.97		267	133	89; 71; 87	
	10.28		267	133	75; 89; 115	
Gallic acid	15.89	169		125		0.78 ± 0.06
Methyl coumarate	17.07	177		103; 131; 59	59	
	28.68	177		103; 131		
Caftaric acid	36.37		623	311;179	149; 179; 135	0.36 ± 0.02
Ethyl cinnamate	40.08	175		129; 85; 157	85; 101	
	41.69	175		129; 85; 157	85; 101	
Coutaric acid	46.50	295		163; 149	119	
	49.27	295		163; 149	119	
Ethyl hydroxybenzoate	71.26	165		147		

(a) Pomegranate peel (present in the Cordoba extract); (b) White Wine; (c) White Wine Vinegar.

The pseudomolecular ion [M−H]^−^ *m/z* 781 was tentatively identified as punicalin due to the MS^2^ fragmentation pattern *m/z* 721, 601 and 299 in agreement with was previously reported^23–25^. Punicalagin was tentatively identified as [M−H]^−^ *m/z* 1083 with MS^2^ fragments *m/z* 781 (punicalin), 601 and 301. The pseudomolecular ion *m/z* 301 corresponds to ellagic acid with MS^2^ fragments *m/z* 257, 229 and 185^20^.

The compounds punicalin, punicalagin and ellagic acid are not present in oak galls, only on the added pomegranate peel^23,25^ and all include at least one pyrogallol moiety with the ability to react with a Fe^2+^ ion. They were all quantified in low amounts. Other gallotannins, like PGG, are also found on pomegranate peel^23,25^.

The recipe Braga mentions the use of white wine vinegar as 33% of the total solvent volume. On the other hand, the recipe Guadalupe mentions the use of white wine as 25% of the total solvent volume, while the recipe Madrid asks for 100%. The phenolic profiles of both white wine vinegar and white wine were analysed by HPLC–ESI–MS and quantified by HPLC–DAD, after the early described liquid–liquid extraction with ethyl acetate and acetonitrile. Table 4b,c show all the organic acids and phenolic compounds present in white wine and white wine vinegar, respectively. They also show the concentration of all identified compounds bearing a catechol or pyrogallol moiety.

The HPLC–ESI–MS analysis to both solvents allowed the tentative identification of pseudomolecular ions: [M−H]^−^ *m/z* 169 corresponding to gallic acid; [M−H]^−^ *m/z* 179 corresponding to caffeic acid since the MS^2^ spectra show a product ion with *m/z* 135; [M−H]^−^ *m/z* 133 corresponding to malic acid since the MS^2^ spectra show fragments with *m/z* 115 and 87; [M−H]^−^ *m/z* 311 was attributed to caftaric acid because the MS^2^ spectra show fragments with *m/z* 179 and 149; [M−H]^−^ *m/z* 295 and its MS^2^ fragment with *m/z* 163 corresponds to coutaric acid; [M−H]^−^ *m/z* 175 corresponding to ethyl cinnamate and [M−H]^−^ *m/z* 177 corresponding to methyl coumarate^26–28^.

The white wine analysis also showed the presence of tartaric and cinnamic acids, ethyl gallate and quercetin glucuronide. Tartaric acid was tentatively identified as the pseudomolecular ion [M−H]^−^ *m/z* 149 with MS^2^ *m/z* 87 and cinnamic acid was tentatively identified due to its pseudomolecular ion [M−H]^−^ *m/z* 147 and respective MS^2^ spectra showing the pseudomolecular ion *m/z* 129. The pseudomolecular ion [M−H]^−^ *m/z* 197 with MS^2^ *m/z* 169 was attributed to ethyl gallate^26^ and the pseudomolecular ion [M−H]^−^ *m/z* 477 was tentatively identified as quercetin glucuronide since the MS^2^ spectra show a pseudomolecular ion with *m/z* 301 ([M−176], loss of a glucuronide moiety) and the MS^3^ spectra show the typical pseudomolecular ions for quercetin^19^. Gallic and sinapic acids with pseudomolecular ions [M−H]^−^ *m/z* 169 and 233 respectively, and ethyl hydroxybenzoate with [M−H]^−^ *m/z* 165, a common antifungal food preservative, were also present in the white wine vinegar^29,30^.

It is also important to refer that, as expected, no tannins (gallotannins and catechins) were detected in both the white wine and vinegar.

To study the effect of “stirring every day with a dried branch from a fig tree” as the recipe Guadalupe demands, the water:white wine (1:0.25) solvent solution was prepared, analysed and stirred with the same dried branch from a fig tree used to prepare the extract. It was possible to find only trace amounts of digalloylglucose, trigalloylglucose, tetragalloylglucose, PGG and gallic acid. However, their concentration is similar to the concentration present in the solvent mixture (water:white wine, 1:0.25) so the action of stirring with the fig tree has no expression in the results (data not shown).

### pH

The pH values measured for all extracts and inks are reported in Table 5. When the recipe demanded to heat the extract after an extraction period, it was recorded the pH before and after heating. For the inks it was again recorded the pH value with and without gum arabic.

**Table 5:** pH values of the extracts (before and after heating) and inks (with and without gum arabic) for all recipes produced.

Recipe	Extract		Ink	
	Before heating	After heating	noGA	GA
Braga		3.14 ± 0.03^c,b^	1.41 ± 0.02^f^	1.39 ± 0.03^g,f^
Montpellier	3.5 ± 0.2^a^	3.7 ± 0.1^d^	1.65 ± 0.04	1.82 ± 0.06
Cordoba	3.11 ± 0.07^b^	3.2 ± 0.1^c^	2.0 ± 0.1^h^	2.1 ± 0.1
Guadalupe	3.61 ± 0.02^a,e^	3.67 ± 0.04^d,e^	1.46 ± 0.08^f^	1.36 ± 0.06^g^
Madrid	3.126 ± 0.009^b^		1.94 ± 0.05^i^	1.98 ± 0.05^h,i^

All pH values with the same letters are significantly equals (P < 0.05).

Heating the extract for the recipes Montpellier, Cordoba and Guadalupe does not contribute to a significant pH variation. The extracts Braga and Cordoba display the lowest pH values, but the recipe Braga uses white wine vinegar as one third of the solvent.

The addition of FeSO_4_ decreases the pH values significantly, especially for the recipe Guadalupe, however, the lowest pH value belongs to the recipe Braga, the recipe that uses the highest amount of FeSO_4_. On the other hand, Cordoba is the ink with the highest pH value. With the addition of gum arabic, the pH remains essentially the same. The binding of iron to the catechol groups may explain these results that lead to deprotonation^12,16^; so, the highest the rate of complex formation, the lowest the pH value expected.

### Overall discussion

The analysis of the data obtained by HPLC–PDA and HPLC–ESI–MS, proved that PGG and HGG are the phenolic compounds present in higher concentration (except in the recipe Cordoba). Overall, it was shown that the percentage of gallic acid in the phenolic extract is higher for the extraction methods in which only water is used, Cordoba and Montpellier. Cordoba recipe, in which the galls are extracted during 8 days at room temperature and then just brought to a boil, was the only ink in which gallic acid was found as the major compound, both in the extracts (65 ± 3%) and the ink (88 ± 1%); this was also the recipe with the lowest extraction yield of phenolic compounds (7 ± 4 mg/mL).

The other three recipes were prepared with other solvents/solvent mixtures: water:vinegar (Braga) and water:wine (Guadalupe) or only wine (Madrid). It was very interesting to observe that, contrarily to what may be expected, wine was not the most efficient extraction method for phenolic compounds (17.8 ± 0.6 mg/mL). The best performing extraction was with the mixture water: wine in the proportion of 1:0.25 (51 ± 4 mg/mL), and even the solution water:vinegar (2:1) achieved better results than using only wine (26 ± 3 mg/mL).

It is important to note that the added extra-solvents (white wine and white wine vinegar) have a few compounds like caffeic, gallic and caftaric acids bearing a catechol or pyrogallol moiety in their constitution and are therefore also able to form or participate in the phenol–metal complex. Further studies are being conducted to understand the formation of these iron-complexes.

Principal component analysis (PCA) models were tested to check for correlations between variables not detected in our analysis of the results, which could disclose consistent patterns that helped us to differentiate and to cluster profiles, providing a systematic analysis of the collected data. However, no other correlations could be disclosed (a summary of the main results is presented as Supplementary Information).

## Conclusions

The results obtained show that the solvent plays a crucial role in the extraction efficiency and in defining the final phenolic profile. When water is mixed with wine (even in low amounts) or vinegar, or when wine is used as the sole solvent, the efficacy is much higher when compared to water, and the major species in solution are polygalloyl esters of glucose that, upon the addition of iron sulfate, will form complexes of Fe^3+^-polygalloyl esters of glucose as dark chromophores. Part of them may grow until forming insoluble organometallic networks^31^, resulting in pigments that are finely dispersed in solution. This phenomenon would explain why the addition of FeSO_4_ decreased the concentration of the phenolic compounds in solution.

This research also proves that iron-gall inks are complex systems that cannot be represented solely by an iron-gallate. Polygalloyl esters of glucose will be the main building blocks for the black colour development, leading to insoluble organometallic networks (pigments) and soluble complexes (dyes). It is also possible that gum arabic, the third vital ingredient, may play a key role in the growth and stability of these organometallic networks. This will be addressed in future research.

For the conservation of cultural heritage, this means that all research strategies focused on the study of gallic acid or tannic acid (decagalloylglucose) as standards for galls, are not considering the molecular structures representative of the colorants of iron gall inks. These data on the accurate characterization of the ligands for iron, used in ancient writing inks, will allow great advances in the study of iron coordination and the factors that affect its stability. If iron is strongly bound to the polygalloyl esters of glucose or encapsulated by gum arabic, it will not move to its support initiating its corrosion. The new knowledge disclosed in this paper will thus pave the way to sustainable conservation strategies for these precious testimonies of our past.

## Materials and methods

### Reagents and solvents

Gallic acid was purchased from Sigma-Aldrich. Gallnuts or “oak apples” from *Quercus infectoria* (batch number: 37400) and gum arabic in grains from *A. senegal* (batch number: 63300) were purchased from Kremer Pigmente. Formic and acetic acid and acetonitrile were obtained from ChemLab. Pomegranate, white wine vinegar (pH 2.54) and white wine (pH 3.33) were purchased in a local supermarket. A branch was taken from a local fig tree.

### Preparation of historic ink reconstructions

The five inks studied were prepared following the medieval treatises written between the fifteenth and seventeenth centuries that were explained elsewhere^12^. Briefly, the galls were grounded or crushed (according to each recipe) using a granite mortar, then weighted and each recipe prepared according to Table 1. Each recipe was reproduced in quintuplicate, and each reproduction analysed in triplicate.

### HPLC–PDA and HPLC–ESI–MS analysis: Gallotannin method

All samples were analysed by HPLC–PDA and HPLC–ESI–MS as reported elsewhere^12^. The analyses were performed in a Finnigan Surveyor Plus HPLC fitted with a PDA Plus detector, an auto-sampler Plus and a LC quaternary pump plus coupled to a Finnigan LCQ Deca XP Plus mass detector equipped with an ESI source and an ion trap quadrupole. The stationary phase was a Thermo Finnigan Hypersil Gold column (150 × 4.6 mm i.d., 5 μm) at 25 °C. The mass spectrometer was operated in the negative-ion mode with source, with a capillary temperature of 275 °C and capillary voltages of 4.5 kV. The mass spectra were recorded between 150 and 2000 *m/z*.

The mobile phases were composed by solvent A, 1% (v/v) formic acid, and solvent B, 100% (v/v) acetonitrile. The flow rate was 0.50 mL/min, the injection volume was 0.25 µL and the gradient method started with a linear gradient ranging from 90% A to 65% A in 50 min, then reaching 100% B in 5 min, and a final isocratic gradient of 100% B during 7 min and a final re-equilibration isocratic gradient of 90% A for 5 min^32^.

### HPLC–DAD analysis: Gallotannin method

The samples were analysed as reported elsewhere^12^. They were performed in a Merck-Hitachi Elite LaChrom HPLC–DAD on a 150 × 4.6 mm i.d., 5 µm pore size reversed-phase C18 column (Merck) thermostated at 25 °C (Merck-Hitachi Column Oven L-2300). Detection was carried out at 280 nm using a diode array detector (Merck-Hitachi Diode Array Detector L-2455). The method used was the same as for HPLC–ESI–MS analysis.

The calibration curve for gallic acid and the study of the repeatability of this method were obtained as reported elsewhere^12,32^. A concentration range of 0.253–0.00253 mg/mL was used and each sample was prepared in duplicate and injected in triplicate. Unknown concentrations were determined from the regression equation (yy = 7 × 10^7^ xx + 70,367; r^2^ = 0.9985) and the results were expressed as mean ± standard deviation and presented as mg/mL equivalents of gallic acid.

### Phenolic compound extraction of white wine, white wine vinegar and pomegranate peel

600 µL of white wine or white wine vinegar were transferred to a microtube and 600 µL of ethyl acetate and 300 µL of acetonitrile were added. The mixture was vortexed for 10 s and then centrifuged at 5400*g* for 5 min^33^. Subsequently, the organic phase was transferred to a new microtube and speed-vacuum dried. This procedure was performed two times in triplicate. The dried residue was re-dissolved in 30 µL of water plus 30 µL of methanol for HPLC analysis.

Lyophilized pomegranate peel sample was prepared following the recipe Cordoba extract preparation without the addition of grounded galls.

### HPLC–ESI–MS and HPLC–DAD analysis: low molecular weight method

The analyses were performed using the same devices and C18 column described earlier. The mobile phase and gradient method were the same as reported elsewhere as low molecular weight HPLC–ESI–MS method^32^. Once again, the HPLC–DAD method used was the same as for the HPLC–ESI–MS analyses.

The calibration curve for gallic acid and the study of the repeatability of this method were obtained as reported elsewhere^12,32^. A concentration range of 0.253–0.00253 mg/mL was used and each sample was prepared in duplicate and injected in triplicate. Unknown concentrations were determined from the regression equation (yy = 1 × 10^8^ xx − 227,051; r^2^ = 0.9993) and the results were expressed as mean ± standard deviation and presented as mg/mL equivalents of gallic acid.

### Statistical analysis

All tests were reproduced in triplicate each and analysed also in triplicate. Values are expressed as the arithmetic means ± standard deviation. Statistical significance performed by one-way analysis of variance (ANOVA) followed by the Tukey's multiple comparisons test with significant difference with 95% confidence interval (P < 0.05), [GP: P > 0.05 (ns), P ≤ 0.05 (*), P ≤ 0.01 (**), P ≤ 0.001 (***), P ≤ 0.0001 (****)], using the software GraphPad Prism 7.2 (San Diego, California, USA).

## Supplementary Information

Supplementary Information.
