# Supplementary material for: In-depth phenolic characterization of iron gall inks by deconstructing representative Iberian recipes
Source: Sci Rep. 2021 Apr 23;11:8811. doi: 10.1038/s41598-021-87969-3 (PMC8065154; doi:10.1038/s41598-021-87969-3)
Supplement: Supplementary file 1 — Supplementary Information. [file 41598_2021_87969_MOESM1_ESM.pdf]

In-depth phenolic characterization of iron gall inks by deconstructing representative Iberian recipes

Natércia Teixeira[1,*], Paula Nabais[2], Victor de Freitas[1], Maria João Melo[2,*]

[1]LAQV/REQUIMTE, Department of Chemistry and Biochemistry, Faculty of Sciences, Universidade do Porto, Rua do Campo Alegre, s/n, 4169-007 Porto, Portugal

[2] LAQV/REQUIMTE, Department of Conservation and Restoration, Faculty of Sciences and Technology, Universidade NOVA de Lisboa, 2829-516 Monte da Caparica, Portugal

*Author to whom correspondence should be addressed: natercia.teixeira@fc.up.pt; mjm@fct.unl.pt

Supporting information

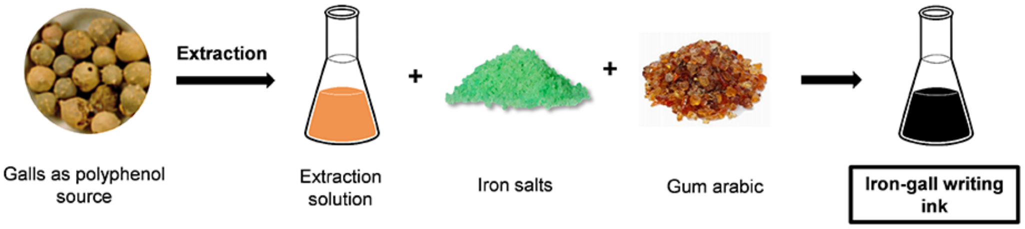

Scheme 1: Main ingredients in the production of iron gall ink.

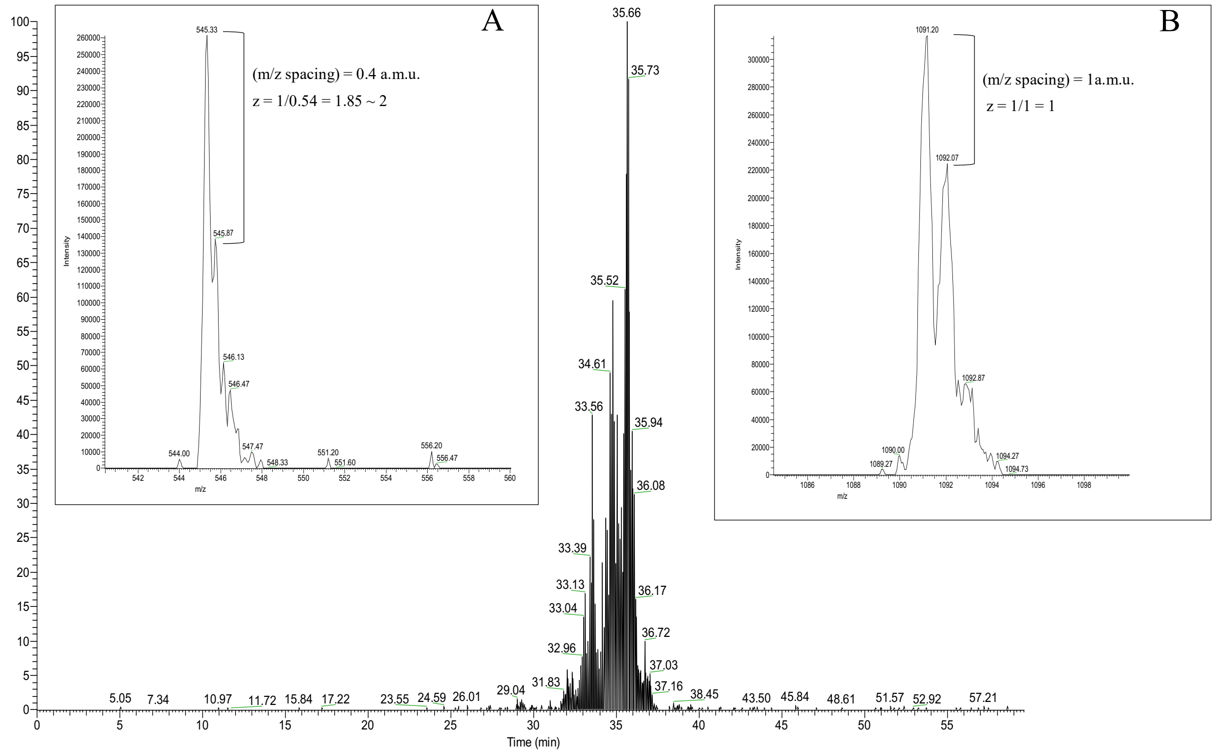

Figure S1: SIM-MS (selected ion monitory – mass spectrometry) chromatogram of Hexagalloylglucose isomers present in the Braga extract (Figure 2 and Table 2). Inset A: $[M-2H]^{2-}$ m/2z 545; Inset B: $[M-H]^{-}$ m/z 1091.

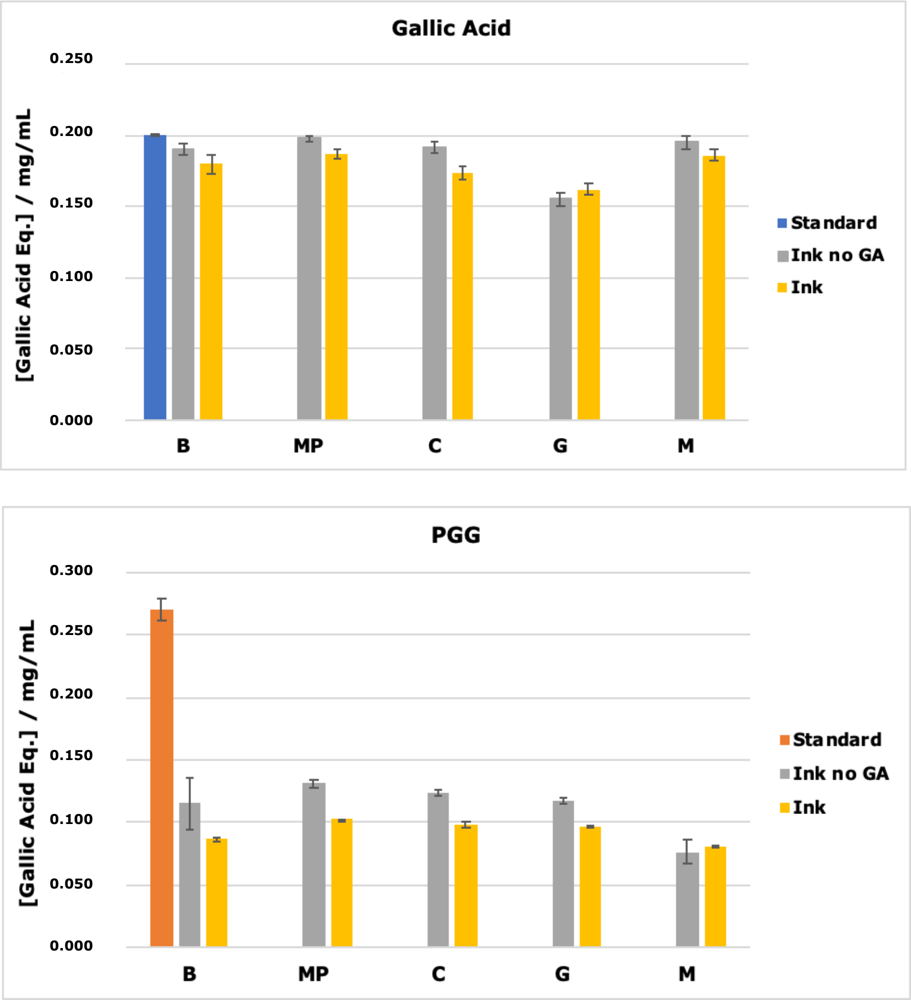

Figure S2: Analysis by HPLC (using the gallotannin method) of the concentration of standard gallic acid and standard PGG after the addition of $FeSO_4$ and gum arabic (GA) maintaining the proportions demanded by each recipe studied and using no heat.

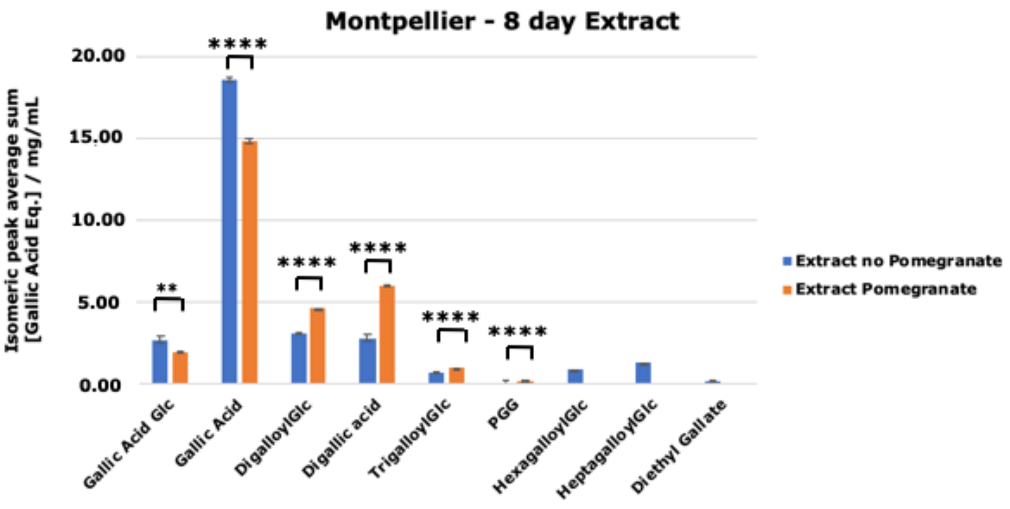

Figure S3: Concentration of all the phenolic compounds (expressed in mg/mL of equivalents of gallic acid) identified in the 8-day Montpellier extracts with and without the addition of pomegranate peel. Statistical significance P < 0.05.

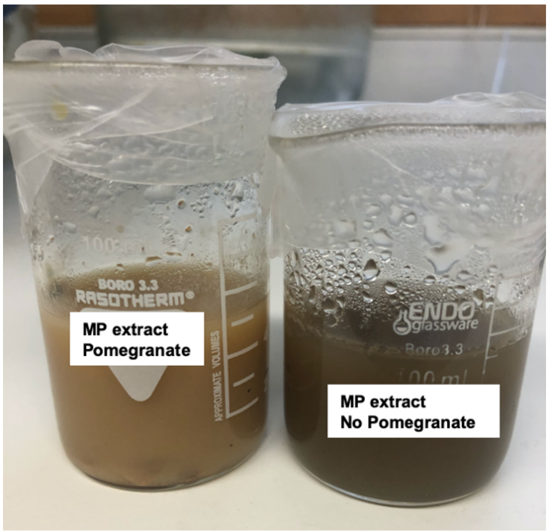

Figure S4: Montpellier (MP) 8-day extract with the addition of pomegranate peel (left) and the same extract without the addition of pomegranate peel (right).

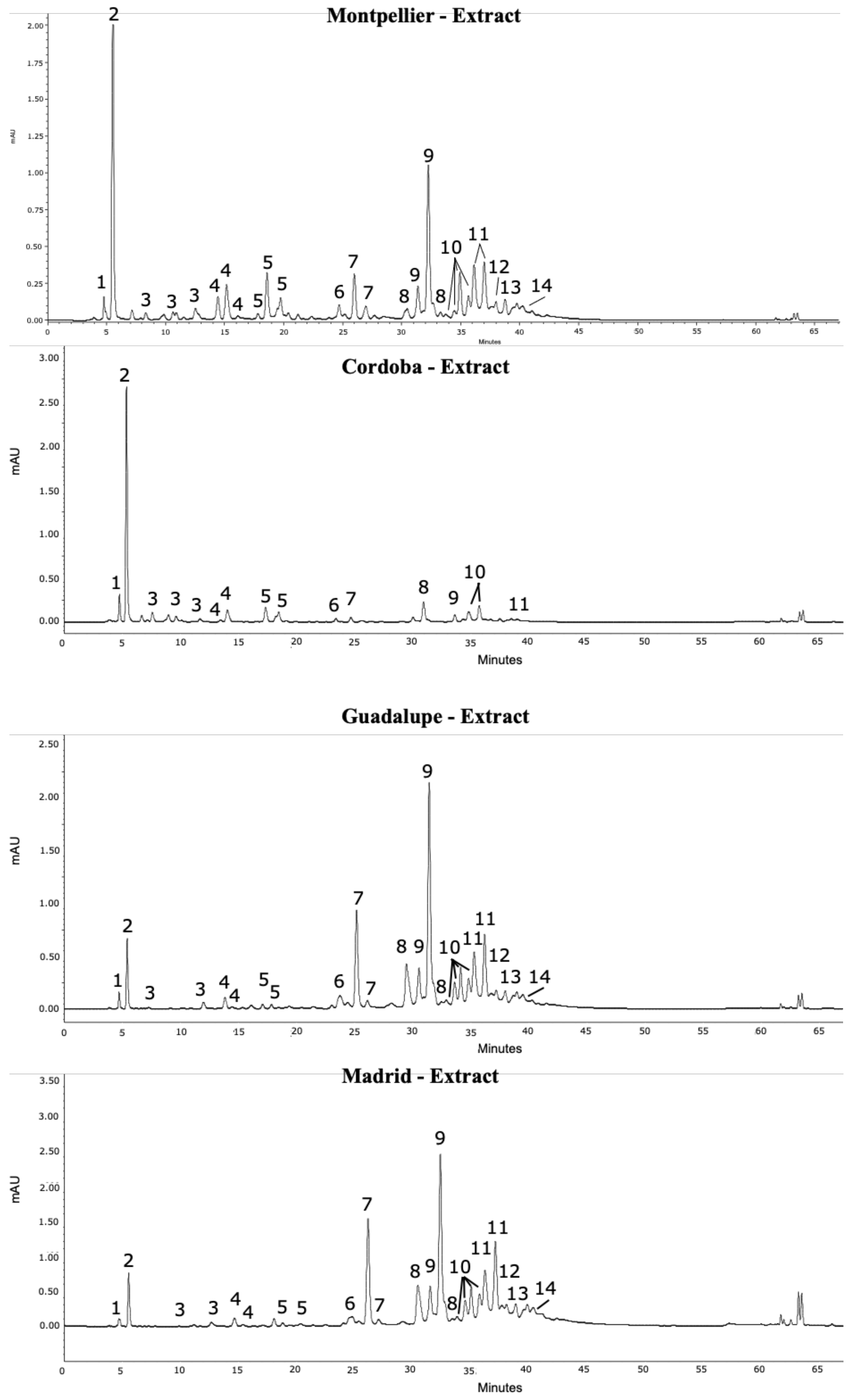

Figure S5: HPLC-ESI-MS chromatogram for the Montpellier, Cordoba, Guadalupe and Madrid extracts, obtained using the gallotannin method (for more details, please see Materials and Methods).

Principal component analysis report

Principal component analysis (PCA) models were developed from Gallic acid, PGG+HGG and sum of phenolic compounds concentrations (Table 3). Models for extracts, inks with and without arabic gum were performed in Matlab Version 8.6 (R2015b) (The Mathworks, Natick, MA) and the PLS Toolbox Version 8.2.1 (Eigenvector Research, Manson, WA). Autoscaling was applied to all three variables included in the PCA model (scaling was applied for extracts, inks with and without arabic gum independently). More than 95% of variance was captured in all three models with two principal components. This can be explained by the correlation found between the sum of phenolic compounds and the PGG+HGG concentrations. The concentration of gallic acid was found to be mainly uncorrelated with the remaining especially in the case of the extracts. The variability found in terms of sum of phenolic compounds and the PGG+HGG concentrations is mainly expressed in all models in the first principal component while the variability of gallic acid is expressed in the second principal component. For instance, Montpellier and Guadalupe recipes show a higher variability in terms of gallic acid concentrations. On the other hand, recipes from Cordoba, despite presenting substantially lower amounts of phenolic compounds and PGG+HGG are also more consistent and present less intrinsic variability. Madrid samples present also very consistent results for all samples (also showing lower amounts of gallic acid), independently of the analyzed dataset. Braga samples present a non-negligeable variability in terms of phenolic compounds and PGG+HGG. Considering the different recipes and the different datasets it is possible in many cases to cluster the results according to the recipe, except probably samples from Braga that show some intrinsic variability, do not forming a noticeable cluster in the first two components.

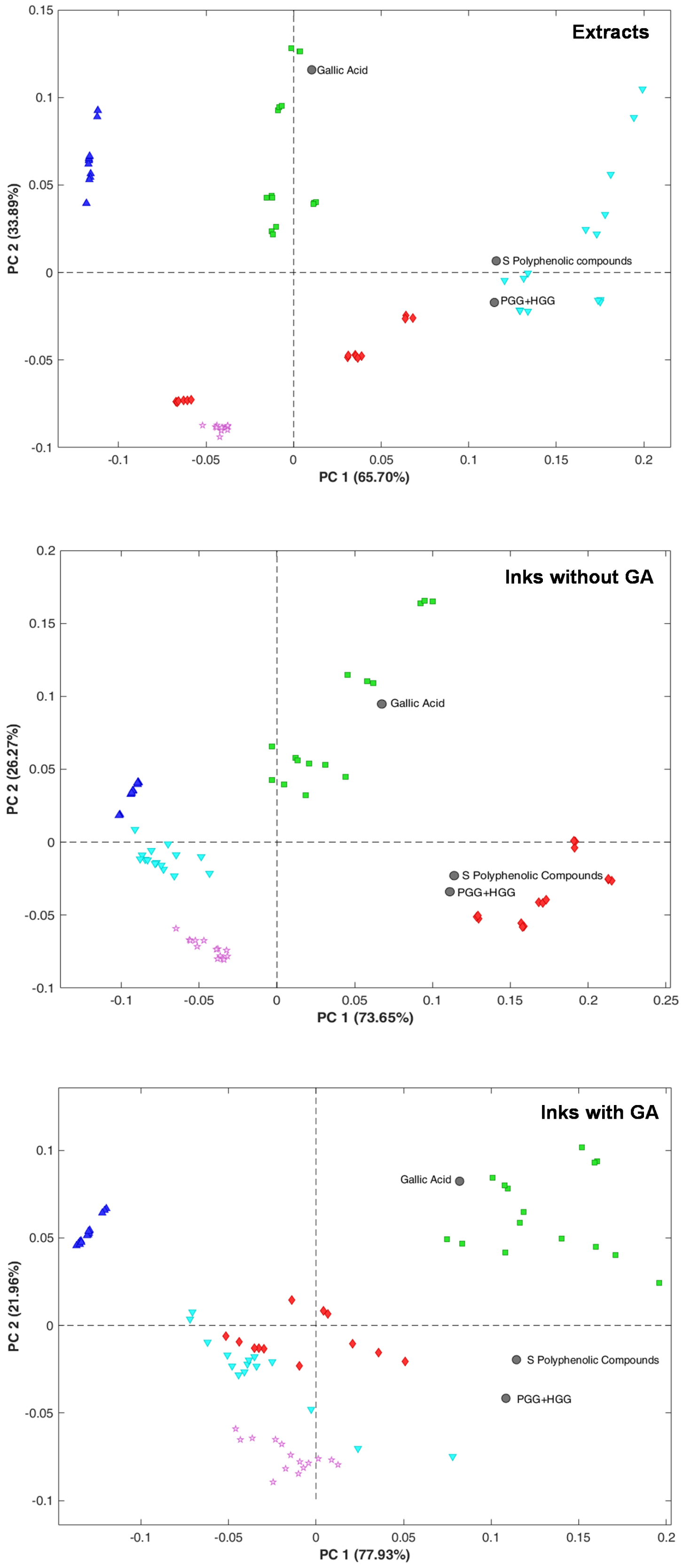

Figure: Principal component analysis biplot for the variables presented in Table 3. ● - correspond to the PC1 and PC2. ◆- Braga scores; ■- Montpellier scores; ▲ - Cordoba scores; ▽ - Guadalupe scores; ☆ - Madrid scores.

Table S1: Concentration of all individual phenols (expressed in mg/mL of equivalents of gallic acid) found in all extracts and inks following the recipe Braga.

Table S1a) Extracts

Recipe	|Gallic Acid Glucoside|	|Gallic Acid|	|Digalloyl glucose|	|Digalloyl glucose|	|Digallic Acid|	|Digallic Acid|	|Digallic Acid|	|Trigalloyl glucose|	|Trigalloyl glucose |	|Trigalloyl glucose |	|Trigallic Acid|	|Tetragalloyl glucose|	|Tetragalloyl glucose|	Tetragalloyl glucose|
**Braga1**	0.226± 0.006	1.32± 0.01	0.031± 0.004	0.038± 0.003	0.349± 0.005	0.718± 0.006	0.10± 0.02	0.09± 0.01	0.194± 0.004	0.08± 0.02	0.55± 0.02	0.233± 0.006	2.39± 0.02	0.354± 0.004
**Braga2**	0.51± 0.01	2.15± 0.01	0.179± 0.002	0.10± 0.01	0.55± 0.02	1.13± 0.01	0.125± 0.006	0.21± 0.01	0.25± 0.01	0.19± 0.01	1.35 ± 0.02	0.48± 0.02	5.01± 0.02	0.59± 0.02
**Braga3**	0.61± 0.02	2.75± 0.03	0.15± 0.01	0.09± 0.02	0.67± 0.03	1.54± 0.03	0.19± 0.02	0.27± 0.02	0.36± 0.03	0.24± 0.02	1.29± 0.06	0.67± 0.04	5.96± 0.03	0.78± 0.03
**Braga4**	0.25± 0.01	1.352± 0.009	0.04± 0.02	0.047± 0.003	0.35± 0.02	0.749± 0.003	0.117± 0.005	0.108± 0.003	0.185± 0.004	0.096± 0.007	0.54± 0.05	0.254± 0.005	2.5635± 0.0005	0.31± 0.01
**Braga5**	0.53± 0.01	2.12± 0.02	0.20± 0.01	0.088± 0.007	0.54± 0.02	1.18± 0.04	0.15± 0.01	0.22± 0.03	0.26± 0.02	0.22± 0.01	1.16± 0.03	0.48± 0.05	5.09± 0.08	0.59± 0.08
**Average**	0.43± 0.01	1.94± 0.01	0.121± 0.009	0.07± 0.01	0.49± 0.02	1.06± 0.02	0.14± 0.01	0.18± 0.01	0.25± 0.01	0.16± 0.01	0.98± 0.03	0.42± 0.02	4.20± 0.03	0.52± 0.03

Recipe	|PGG|	|PGG|	|PGG|	|PGG|	|Hexagalloyl glucose|	|Hexagalloyl glucose|	|Hexagalloyl glucose|	|Hexagalloyl glucose|	|Hexagalloyl glucose|	|Hexagalloylgl ucose|	|Heptagalloyl glucose |	|Heptagalloyl glucose |	|Diethyl gallate|	**Sum**
**Braga1**	1.67± 0.04	0.606± 0.008	2.61± 0.03	0.038± 0.003	0.08± 0.02	0.45± 0.03	0.614± 0.005	0.293± 0.005	0.59± 0.04	0.881± 0.004	0.21± 0.01	0.038± 0.009	0.072± 0.008	14.8± 0.01
**Braga2**	3.75± 0.08	1.35± 0.04	6.03± 0.04	0.116± 0.0007	0.20± 0.01	1.11± 0.01	1.34± 0.01	0.67± 0.03	1.46± 0.02	2.27± 0.02	0.49± 0.03	0.11± 0.01	0.18± 0.03	31,9±0.02
**Braga3**	4.11± 0.08	1.53± 0.05	7.20± 0.03	0.134± 0.0006	0.19± 0.01	1.20± 0.04	1.43± 0.08	0.75± 0.05	1.61± 0.06	2.53± 0.03	0.52± 0.06	0.13± 0.03	0.23± 0.01	37.1±0.03
**Braga4**	1.72± 0.06	0.68± 0.02	2.96± 0.02	0.052± 0.0006	0.08± 0.02	0.44± 0.08	0.657± 0.004	0.29± 0.03	0.62± 0.07	0.96± 0.09	0.21± 0.03	0.058± 0.005	0.086± 0.002	15.8±0.02
**Braga5**	3.37± 0.03	1.25± 0.08	6.4± 0.1	0.128± 0.0002	0.18± 0.01	1.02± 0.02	1.19± 0.05	0.61± 0.02	1.42± 0.03	2.29± 0.06	0.50± 0.02	0.128± 0.007	0.20± 0.02	31.5±0.04
**Average**	2.925± 0.06	1.08± 0.04	5.03± 0.05	0.094± 0.005	0.15± 0.01	0.84± 0.04	1.05± 0.03	0.52± 0.03	1.14± 0.04	1.79± 0.04	0.39± 0.03	0.09± 0.01	0.15± 0.01	26.22±0.03

Table S1b) Inks without gum arabic

Recipe	|Gallic Acid Glucoside|	|Gallic Acid|	|Digalloyl glucose|	|Digalloyl glucose|	|Digallic Acid|	|Digallic Acid|	|Digallic Acid|	|Trigalloyl glucose|	|Trigalloyl glucose |	|Trigalloyl glucose |	|Trigallic Acid|	|Tetragalloyl glucose|	|Tetragalloyl glucose|	Tetragalloyl glucose|
**Braga1**	0.56± 0.01	3,53± 0.04	0.076± 0.009	0.09± 0.01	0.71± 0.03	1,77± 0.02	0.23± 0.02	0.228± 0.008	0.449± 0.007	0.17± 0.02	0.77± 0.02	0.489± 0.004	4.46± 0.07	0.61± 0.03
**Braga2**	0.52± 0.02	2.37± 0.01	0.11± 0.01	0.08± 0.02	0.46± 0.02	1.15± 0.02	0.1132± 0.0007	0.17± 0.03	0.256± 0.004	0.14± 0.01	0,65 ± 0.08	0.36± 0.02	3.79± 0.05	0.44± 0.02
**Braga3**	0.598± 0.006	2.80± 0.03	0.127± 0.009	0.09± 0.01	0.59± 0.03	1.46± 0.03	0.146± 0.004	0.20± 0.03	0.30± 0.01	0.19± 0.02	0.6± 0.1	0.519± 0.002	4.350± 0.007	0.51± 0.04
**Braga4**	0.61± 0.01	3.305± 0.002	0.09± 0.03	0.09± 0.02	0.71± 0.03	1.68± 0.03	0.17± 0.02	0.24± 0.02	0.387± 0.009	0.20± 0.01	0.76± 0.06	0.54± 0.03	4.70± 0.05	0.62± 0.02
**Braga5**	0.62± 0.01	2.47± 0.02	0.17± 0.02	0.09± 0.02	0.51± 0.01	1.205± 0.007	0.10± 0.01	0.211± 0.004	0.243± 0.009	0.15± 0.01	0.64± 0.03	0.42± 0.01	4.09± 0.04	0.46± 0.01
**Average**	0.58± 0.01	2.90± 0.02	0.11± 0.02	0.09± 0.02	0.60± 0.03	1.45± 0.02	0.15± 0.01	0.21± 0.02	0.327± 0.009	0.17± 0.02	0.69± 0.06	0.47± 0.01	4.28± 0.04	0.53± 0.02

Recipe	|PGG|	|PGG|	|PGG|	|PGG|	|Hexagalloyl glucose|	|Hexagalloyl glucose|	|Hexagalloyl glucose|	|Hexagalloyl glucose|	|Hexagalloyl glucose|	|Hexagalloylglucose|	|Heptagalloyl glucose |	|Heptagalloyl glucose |	|Diethyl gallate|	**Sum**
**Braga1**	2.47± 0.05	1.25± 0.01	6.16± 0.05	0.09± 0.02	0.138± 0.003	0.55± 0.03	1.378± 0.005	0.54± 0.04	1.49± 0.04	2.12± 0.04	0.46± 0.03	0.10± 0.02	0.17± 0.02	31.1±0.03
**Braga2**	2.28± 0.03	1.07± 0.01	5.60± 0.03	0.06± 0.02	0.09± 0.03	0.51± 0.04	1.16± 0.09	0.510± 0.007	1.39± 0.02	2.00± 0.09	0.41± 0.04	0.114± 0.005	0.18± 0.01	26.0±0.03
**Braga3**	2.33± 0.05	1.18± 0.05	6.7± 0.1	0.063± 0.007	0.10± 0.01	0.48± 0.03	1.23± 0.08	0.58± 0.01	1.5± 0.1	2.37± 0.05	0.44± 0.05	0.10± 0.02	0.19± 0.02	29.8±0.04
**Braga4**	2.7± 0.1	1.34± 0.02	7.09± 0.03	0.091± 0.009	0.146± 0.005	0.58± 0.09	1.52± 0.01	0.65± 0.01	1.769± 0.002	2.58± 0.09	0.56± 0.03	0.137± 0.008	0.21± 0.04	33.4±0.03
**Braga5**	2.21± 0.01	1.12± 0.03	6.71± 0.02	0.067± 0.002	0.08± 0.02	0.51± 0.03	1.27± 0.01	0.51± 0.04	1.627± 0.007	2.445± 0.007	0.43± 0.04	0.10± 0.03	0.17± 0.06	28.6±0.02
**Average**	2.39± 0.05	1.19± 0.02	6.46± 0.06	0.07± 0.01	0.11± 0.01	0.53± 0.04	1.31± 0.04	0.56± 0.02	1.56± 0.03	2.30± 0.06	0.46± 0.04	0.11± 0.02	0.18± 0.03	29.78±0.03

Table S1c) Inks with gum arabic

Recipe	|Gallic Acid Glucoside|	|Gallic Acid|	|Digalloyl glucose|	|Digalloyl glucose|	|Digallic Acid|	|Digallic Acid|	|Digallic Acid|	|Trigalloyl glucose|	|Trigalloyl glucose |	|Trigalloyl glucose |	|Trigallic Acid|	|Tetragalloyl glucose|	|Tetragalloyl glucose|	Tetragalloyl glucose|
**Braga1**	0.506± 0.004	3,16± 0.04	0.035± 0.005	0.111± 0.003	0.59± 0.02	1.36± 0.02	0.125± 0.005	0.134± 0.008	0.28± 0.03	0.05± 0.01	0.32± 0.04	0.26± 0.02	2.38± 0.01	0.27± 0.02
**Braga2**	0.377± 0.005	2.04± 0.01	0.032± 0.003	0.05± 0.03	0.26± 0.03	0.70± 0.03	0.030± 0.007	0.042± 0.008	0.09± 0.02	0.021± 0.001	0.03 ± 0.02	0.09± 0.03	0.78± 0.01	0.07± 0.02
**Braga3**	0405± 0.006	1.61± 0.03	0.059± 0.007	0.047± 0.009	0.162± 0.005	0.47± 0.02	0.004± 0.002	0.012± 0.007	0.032± 0.002	0.009± 0.002	0.014± 0.005	0.10± 0.02	0.52± 0.07	0.055± 0.005
**Braga4**	0.407± 0.005	2.11± 0.002	0.034± 0.004	0.07± 0.03	0.23± 0.02	0.62± 0.02	0.011± 0.003	0.036± 0.007	0.04± 0.02	0.010± 0.002	0.006± 0.005	0.075± 0.009	0.34± 0.06	0.03± 0.01
**Braga5**	0.39± 0.01	1.58± 0.02	0.052± 0.004	0.025± 0.002	0.125± 0.002	0.40± 0.04	<LOQ	0.017± 0.006	<LOQ	0.004± 0.001	0.003± 0.003	0.04± 0.01	0.18± 0.01	0.02± 0.01
**Average**	0.416± 0.006	2.10± 0.02	0.042± 0.005	0.06± 0.02	0.27± 0.02	0.71± 0.03	0.043± 0.004	0.048± 0.007	0.11± 0.02	0.018± 0.003	0.07± 0.02	0.11± 0.02	0.84± 0.03	0.09± 0.01

Recipe	|PGG|	|PGG|	|PGG|	|PGG|	|Hexagalloyl glucose|	|Hexagalloyl glucose|	|Hexagalloyl glucose|	|Hexagalloyl glucose|	|Hexagalloyl glucose|	|Hexagalloylglucose|	|Heptagalloyl glucose |	|Heptagalloyl glucose |	|Diethyl gallate|	**Sum**
**Braga1**	0.9± 0.1	0.76± 0.04	4.88± 0.01	0.046± 0.002	0.036± 0.004	0.13± 0.03	1.00± 0.08	0.30± 0.05	1.16± 0.06	1.69± 0.05	0.25± 0.01	0.081± 0.003	0.12± 0.03	20.9±0.03
**Braga2**	0.32± 0.03	0.27± 0.03	2.46± 0.02	0.009± 0.001	<LOQ	0.06± 0.05	0.42± 0.05	0.11± 0.02	0.70± 0.04	0.91± 0.07	0.09± 0.03	0.027± 0.004	0.05± 0.02	10.0±0.02
**Braga3**	0.26± 0.02	0.20± 0.04	1.90± 0.01	0.001± 0.001	<LOQ	0.04± 0.01	0.24± 0.06	0.07± 0.01	0.49± 0.08	0.7± 0.1	0.06± 0.02	0.010± 0.004	0.026± 0.007	7.45±0.02
**Braga4**	0.11± 0.03	0.23± 0.02	1.91± 0.01	0.0027± 0.0003	<LOQ	0.009± 0.007	0.26± 0.03	0.06± 0.01	0.60± 0.08	0.78± 0.06	0.05± 0.02	0.009± 0.003	0.028± 0.005	8.06±0.02
**Braga5**	0.09± 0.04	0.10± 0.03	2.37± 0.09	0.0007± 0.0001	<LOQ	0.007± 0.004	0.155± 0.004	0.027± 0.005	0.28± 0.06	0.3± 0.1	0.02± 0.01	0.003± 0.004	0.01± 0.01	6.27±0.02
**Average**	0.33± 0.05	0.31± 0.03	2.70± 0.03	0.012± 0.001	0.036± 0.004	0.05± 0.02	0.42± 0.04	0.11± 0.02	0.65± 0.07	0.87± 0.08	0.10± 0.02	0.026± 0.003	0.05± 0.01	10.55±0.02

Table S2: Concentration of all individual phenols (expressed in mg/mL of equivalents of gallic acid) found in all extracts and inks following the recipe Montpellier.

Table S2a) Extracts before heating

Recipe	|Gallic Acid Glucoside|	|Gallic Acid|	|Digalloyl glucose|	|Digalloyl glucose|	|Digallic Acid|	|Digallic Acid|	|Digallic Acid|	|Trigalloyl glucose|	|Trigalloyl glucose |	|Trigalloyl glucose |	|Trigalloyl glucose |	|Trigallic Acid|	|Tetragalloyl glucose|
**Montpellier1**	0.373± 0.003	5.36± 0.01	0.35± 0.01	0.065± 0.002	0.15± 0.02	0.059± 0.005	0.0079± 0.0001	0.57± 0.05	<LOQ	0.74± 0.01	0.65± 0.02	0.135± 0.008	0.04± 0.01
**Montpellier2**	0.139± 0.009	2.57± 0.01	0.039± 0.004	0.071± 0.006	0.101± 0.006	0.035± 0.009	0.139± 0.002	0.39± 0.01	<LOQ	0.48± 0.02	0.12 ± 0.02	0.18± 0.02	0.137± 0.002
**Montpellier3**	0.165± 0.001	3.41± 0.03	0.06± 0.01	0.057± 0.007	0.14± 0.01	0.018± 0.006	0.06± 0.05	0.590± 0.0005	<LOQ	0.678± 0.004	0.34± 0.01	0.15± 0.01	0.223± 0.006
**Montpellier4**	0.156± 0.003	3.5± 0.009	0.06± 0.01	0.06± 0.02	0.16± 0.01	0.037± 0.005	0.049± 0.009	0.64± 0.03	<LOQ	0.71± 0.02	0.38± 0.02	0.11± 0.01	0.21± 0.01
**Montpellier5**	0.254± 0.003	5.75± 0.02	0.18± 0.01	0.147± 0.001	0.205± 0.006	0.07± 0.01	0.0177± 0.0003	0.59± 0.02	<LOQ	0.88± 0.03	0.66± 0.06	0.13± 0.01	0.06± 0.01
**Average**	0.217± 0.004	4.12± 0.01	0.14± 0.01	0.079± 0.007	0.15± 0.01	0.043± 0.007	0.06± 0.01	0.55± 0.02	--	0.70± 0.02	0.43± 0.03	0.14± 0.01	0.134± 0.009

Recipe	Tetragalloyl glucose|	|PGG|	|PGG|	|PGG|	|Hexagalloyl glucose|	|Hexagalloyl glucose|	|Hexagalloyl glucose|	|Hexagalloyl glucose|	|Hexagalloyl glucose|	|Heptagalloyl glucose |	|Diethyl gallate|	**Sum**
**Montpellier1**	0.12± 0.01	0.22± 0.01	0.252± 0.005	2.0± 0.1	0.03± 0.01	0.44± 0.03	0.0011± 0.0003	0.13± 0.01	0.29± 0.03	0.206± 0.006	0.046± 0.003	12.26±0.02
**Montpellier2**	0.121± 0.009	0.229± 0.009	0.30± 0.01	2.09± 0.05	0.044± 0.003	0.52± 0.02	0.170± 0.005	0.64± 0.02	0.851± 0.004	0.19± 0.01	0.055± 0.005	9.60±0.01
**Montpellier3**	0.122± 0.008	0.116± 0.002	0.24± 0.02	1.91± 0.06	0.03± 0.01	0.46± 0.02	0.14± 0.02	0.684± 0.004	0.89± 0.01	0.199± 0.005	0.060± 0.009	10.74±0.01
**Montpellier4**	0.12± 0.01	0.088± 0.009	0.24± 0.01	1.76± 0.06	0.037± 0.001	0.44± 0.03	0.152± 0.005	0.64± 0.05	0.867± 0.009	0.18± 0.02	0.064± 0.008	10.67±0.02
**Montpellier5**	0.05± 0.01	0.224± 0.007	0.22± 0.01	1.62± 0.02	0.0374± 0.0003	0.433± 0.008	0.068± 0.002	0.551± 0.005	0.74± 0.06	0.169± 0.001	0.04± 0.01	13.10±0.02
**Average**	0.11± 0.01	0.176± 0.008	0.25± 0.01	1.88± 0.06	0.036± 0.005	0.46± 0.02	0.106± 0.007	0.53± 0.02	0.73± 0.02	0.189± 0.009	0.053± 0.007	11.27±0.02

Table S2b) Extracts after heating

Recipe	|Gallic Acid Glucoside|	|Gallic Acid|	|Digalloyl glucose|	|Digalloyl glucose|	|Digallic Acid|	|Digallic Acid|	|Digallic Acid|	|Trigalloyl glucose|	|Trigalloyl glucose |	|Trigalloyl glucose |	|Trigalloyl glucose |	|Trigallic Acid|	|Tetragalloyl glucose|
**Montpellier1**	1.08± 0.04	5.71± 0.03	0.286± 0.008	0.25± 0.01	0.63± 0.04	0.31± 0.01	0.522± 0.006	2.08± 0.009	0.066± 0.004	1.61± 0.03	1.14± 0.02	0.388± 0.009	1.38± 0.02
**Montpellier2**	2.4± 0.1	4.37± 0.01	0.36± 0.03	0.286± 0.009	0.58± 0.04	0.43± 0.02	0.61± 0.02	1.83± 0.01	0.12± 0.01	1.94± 0.05	0.96 ± 0.08	0.29± 0.02	1.19± 0.08
**Montpellier3**	2.4± 0.1	4.36± 0.02	0.21± 0.01	0.20± 0.02	0.452± 0.007	0.67± 0.02	0.44± 0.03	0.87± 0.004	0.223± 0.03	0.98± 0.04	0.17± 0.03	0.51± 0.02	1.51± 0.04
**Montpellier4**	2.28± 0.05	3.89± 0.05	0.272± 0.002	0.25± 0.03	0.42± 0.04	0.49± 0.03	0.75± 0.06	1.40± 0.02	0.15± 0.004	1.29± 0.02	0.37± 0.02	0.35± 0.02	1.5± 0.1
**Montpellier5**	3.45± 0.02	6.54± 0.02	0.30± 0.02	0.32± 0.01	0.86± 0.04	0.37± 0.05	0.29± 0.06	2.7± 0.02	0.07± 0.02	2.32± 0.05	0.17± 0.01	0.18± 0.03	1.27± 0.02
**Average**	2.31± 0.06	4.97± 0.03	0.29± 0.01	0.26± 0.02	0.59± 0.03	0.45± 0.03	0.52± 0.04	1.78± 0.01	0.12± 0.01	1.63± 0.04	0.56± 0.04	0.34± 0.02	1.28± 0.06

Recipe	Tetragalloyl glucose|	|PGG|	|PGG|	|PGG|	|Hexagalloyl glucose|	|Hexagalloyl glucose|	|Hexagalloyl glucose|	|Hexagalloyl glucose|	|Hexagalloyl glucose|	|Heptagalloyl glucose |	|Diethyl gallate|	**Sum**
**Montpellier1**	0.44± 0.01	0.60± 0.01	0.79± 0.05	3.92± 0.05	0.15± 0.01	1.05± 0.02	0.33± 0.01	1.47± 0.05	1.90± 0.03	0.4± 0.1	0.130± 0.008	26.55±0.03
**Montpellier2**	0.42± 0.03	0.45± 0.07	0.78± 0.05	3.9± 0.1	0.13± 0.03	1.130± 0.006	0.35± 0.01	1.48± 0.01	1.68± 0.02	0.45± 0.01	0.10± 0.02	26.23±0.04
**Montpellier3**	0.63± 0.06	0.73± 0.01	1.23± 0.07	5.69± 0.02	0.4± 0.1	1.63± 0.02	0.57± 0.02	1.84± 0.03	1.99± 0.04	0.58± 0.02	0.132± 0.009	28.39±0.03
**Montpellier4**	0.523± 0.008	0.50± 0.04	0.91± 0.04	4.59± 0.03	0.11± 0.05	1.28± 0.04	0.39± 0.07	1.59± 0.04	1.69± 0.08	0.49± 0.02	0.11± 0.02	25.61±0.04
**Montpellier5**	0.31± 0.03	0.18± 0.03	0.72± 0.04	3.89± 0.02	0.013± 0.002	1.03± 0.04	0.30± 0.03	1.69± 0.01	1.97± 0.01	0.49± 0.01	0.126± 0.006	29.59±0.03
**Average**	0.46± 0.03	0.49± 0.03	0.89± 0.05	4.40± 0.05	0.16± 0.04	1.22± 0.03	0.39± 0.03	1.61± 0.03	1.85± 0.04	0.47± 0.03	0.12± 0.01	27.27±0.03

Table S2c) Inks without gum arabic

Recipe	|Gallic Acid Glucoside|	|Gallic Acid|	|Digalloyl glucose|	|Digalloyl glucose|	|Digallic Acid|	|Digallic Acid|	|Digallic Acid|	|Trigalloyl glucose|	|Trigalloyl glucose |	|Trigalloyl glucose |	|Trigalloyl glucose |	|Trigallic Acid|	|Tetragalloyl glucose|
**Montpellier1**	0.57± 0.01	4.36± 0.04	0.06± 0.01	0.14± 0.02	0,376± 0.004	0.19± 0.03	0.39± 0.02	0.74± 0.05	0.03± 0.02	0.098± 0.005	0.25± 0.02	0.09± 0.02	0.5± 0.1
**Montpellier2**	0.333± 0.005	2.94± 0.01	0.04± 0.01	<LOQ	0.13± 0.01	0.26± 0.02	0.65± 0.05	0.097± 0.009	0.062± 0.002	0.15± 0.02	0,009± 0.003	0.05± 0.01	0.71± 0.03
**Montpellier3**	0.386± 0.009	3.29± 0.03	0.049± 0.005	<LOQ	0.15± 0.01	0.329± 0.006	0.795± 0.009	0.090± 0.007	0.066± 0.005	0.155± 0.004	0.0085± 0.0004	0.047± 0.007	0.54± 0.02
**Montpellier4**	0.30± 0.02	3.30± 0.002	0.05± 0.02	<LOQ	0.110± 0.004	0.09± 0.03	0.370± 0.008	0.619± 0.002	0.004± 0.001	0.053± 0.002	0.19± 0.03	0.07± 0.02	0.63± 0.03
**Montpellier5**	0.45± 0.01	5.45± 0.02	0.037± 0.003	<LOQ	0.141± 0.002	0.06± 0.01	0.35± 0.02	0.66± 0.02	0.0019± 0.0005	0.05± 0.01	0.47± 0.01	0.10± 0.02	0.49± 0.03
**Average**	0.41± 0.01	3.87± 0.02	0.047± 0.009	0.14± 0.02	0.183± 0.007	0.19± 0.02	0.51± 0.02	0.44± 0.02	0.033± 0.005	0.102± 0.008	0.19± 0.01	0.07± 0.02	0.57± 0.05

Recipe	Tetragalloyl glucose|	|PGG|	|PGG|	|PGG|	|Hexagalloyl glucose|	|Hexagalloyl glucose|	|Hexagalloyl glucose|	|Hexagalloyl glucose|	|Hexagalloyl glucose|	|Heptagalloyl glucose |	|Diethyl gallate|	**Sum**
**Montpellier1**	0.10± 0.04	0.16± 0.09	0.41± 0.05	2.57± 0.02	0.022± 0.001	0.55± 0.09	0.11± 0.03	0.98± 0.05	1.2± 0.1	0.13± 0.08	0.05± 0.01	14.02±0.04
**Montpellier2**	0.04± 0.04	0.3± 0.1	0.37± 0.07	2.60± 0.03	0.04± 0.04	0.5± 0.1	0.12± 0.04	0.83± 0.05	0.82± 0.08	0.13± 0.04	0.029± 0.004	11.29±0.03
**Montpellier3**	0.04± 0.02	0.2± 0.1	1.3± 0.1	2.6± 0.1	0.03± 0.01	0.49± 0.07	0.07± 0.06	0.89± 0.04	0.8± 0.1	0.12± 0.02	0.025± 0.003	12.47±0.03
**Montpellier4**	0.08± 0.04	0.34± 0.02	0.4± 0.1	2.42± 0.04	0.04± 0.04	0.5± 0.1	0.11± 0.04	0.8± 0.1	0.82± 0.02	0.12± 0.03	0.03± 0.01	11.41±0.03
**Montpellier5**	0.086± 0.008	0.34± 0.05	0.48± 0.03	3.17± 0.07	0.029± 0.008	0.64± 0.03	0.152± 0.009	1.09± 0.08	1.11± 0.09	0.20± 0.01	0.04± 0.01	15.60±0.02
**Average**	0.07± 0.03	0.19± 0.08	0.58± 0.07	2.67± 0.06	0.03± 0.02	0.54± 0.08	0.11± 0.04	0.92± 0.07	0.95± 0.08	0.14± 0.04	0.035± 0.009	12.96±0.03

Table S2d) Inks with gum arabic

<table>
<tr><th>Recipe</th><th>|Gallic Acid Glucoside|</th><th>|Gallic Acid|</th><th>|Digalloyl glucose|</th><th>|Digalloyl glucose|</th><th>|Digallic Acid|</th><th>|Digallic Acid|</th><th>|Digallic Acid|</th><th>|Trigalloyl glucose|</th><th>|Trigalloyl glucose |</th><th>|Trigalloyl glucose |</th><th>|Trigalloyl glucose |</th><th>|Trigallic Acid|</th><th>|Tetragalloyl glucose|</th></tr>
<tr><td>Montpellier1</td><td>0.50± 0.06</td><td>4.31± 0.04</td><td>0.11± 0.02</td><td><LOQ</td><td>0.010± 0.004</td><td>0.21± 0.01</td><td>0.14± 0.08</td><td>0.43± 0.02</td><td>0.008± 0.002</td><td>0.12± 0.008</td><td>0.8± 0.1</td><td>0.26± 0.01</td><td>0.7± 0.1</td></tr>
<tr><td>Montpellier2</td><td>0.342± 0.002</td><td>3.76± 0.01</td><td>0.076± 0.009</td><td><LOQ</td><td>0.034± 0.002</td><td>0.15± 0.01</td><td>0.17± 0.03</td><td>0.520± 0.007</td><td><LOQ</td><td>0.092± 0.003</td><td>0.25 ± 0.02</td><td>0.09± 0.008</td><td>0.97± 0.05</td></tr>
<tr><td>Montpellier3</td><td>0.32± 0.05</td><td>3.19± 0.07</td><td>0.045± 0.004</td><td><LOQ</td><td>0.019± 0.002</td><td>0.13± 0.01</td><td>0.24± 0.09</td><td>0.6± 0.1</td><td><LOQ</td><td>0.07± 0.03</td><td>0.15± 0.01</td><td>0.10± 0.01</td><td>0.9± 0.1</td></tr>
<tr><td>Montpellier4</td><td>0.377± 0.009</td><td>3.59± 0.05</td><td>0.061± 0.006</td><td><LOQ</td><td>0.029± 0.002</td><td>0.163± 0.002</td><td>0.24± 0.02</td><td>0.61± 0.03</td><td><LOQ</td><td>0.10± 0.009</td><td>0.20± 0.01</td><td>0.075± 0.02</td><td>1.3± 0.1</td></tr>
<tr><td>Montpellier5</td><td>0.43± 0.01</td><td>3.68± 0.02</td><td>0.090± 0.006</td><td><LOQ</td><td>0.046± 0.005</td><td>0.21± 0.01</td><td>0.22± 0.02</td><td>0.58± 0.01</td><td><LOQ</td><td>0.107± 0.01</td><td>0.45± 0.01</td><td>0.04± 0.009</td><td>1.2± 0.1</td></tr>
<tr><td>Average</td><td>0.39± 0.03</td><td>3.71± 0.04</td><td>0.077± 0.009</td><td>--</td><td>0.028± 0.003</td><td>0.17± 0.01</td><td>0.20± 0.05</td><td>0.55± 0.03</td><td>0.008± 0.002</td><td>0.10± 0.01</td><td>0.37± 0.03</td><td>0.11± 0.01</td><td>1.01± 0.09</td></tr>
</table>

<table>
<tr><th>Recipe</th><th>Tetragalloyl glucose|</th><th>|PGG|</th><th>|PGG|</th><th>|PGG|</th><th>|Hexagalloyl glucose|</th><th>|Hexagalloyl glucose|</th><th>|Hexagalloyl glucose|</th><th>|Hexagalloyl glucose|</th><th>|Hexagalloyl glucose|</th><th>|Heptagalloyl glucose |</th><th>|Diethyl gallate|</th><th>Sum</th></tr>
<tr><td>Montpellier1</td><td>0.16± 0.05</td><td>0.4± 0.1</td><td>0.44± 0.02</td><td>2.5± 0.1</td><td>0.06± 0.05</td><td>0.58± 0.02</td><td>0.16± 0.03</td><td>0.96± 0.04</td><td>1.1± 0.1</td><td>0.19± 0.03</td><td>0.04± 0.01</td><td>13.90±0.04</td></tr>
<tr><td>Montpellier2</td><td>0.13± 0.02</td><td>0.64± 0.03</td><td>0.37± 0.02</td><td>2.06± 0.06</td><td>0.121± 0.008</td><td>0.48± 0.02</td><td>0.155± 0.008</td><td>0.66± 0.03</td><td>0.70± 0.05</td><td>0.157± 0.009</td><td>0.03± 0.01</td><td>11.91±0.02</td></tr>
<tr><td>Montpellier3</td><td>0.11± 0.05</td><td>0.6± 0.1</td><td>0.37± 0.02</td><td>2.3± 0.1</td><td>0.09± 0.04</td><td>0.51± 0.06</td><td>0.132± 0.007</td><td>0.8± 0.1</td><td>0.7± 0.1</td><td>0.14± 0.02</td><td>0.025± 0.004</td><td>11.48±0.05</td></tr>
<tr><td>Montpellier4</td><td>0.17± 0.02</td><td>0.87± 0.09</td><td>0.44± 0.06</td><td>2.4± 0.1</td><td>0.15± 0.03</td><td>0.52± 0.04</td><td>0.16± 0.02</td><td>0.68± 0.07</td><td>0.80± 0.03</td><td>0.17± 0.02</td><td>0.022± 0.007</td><td>13.06±0.03</td></tr>
<tr><td>Montpellier5</td><td>0.21± 0.05</td><td>0.8± 0.1</td><td>0.56± 0.06</td><td>3.2± 0.1</td><td>0.12± 0.03</td><td>0.77± 0.07</td><td>0.17± 0.04</td><td>1.00± 0.08</td><td>1.13± 0.04</td><td>0.24± 0.03</td><td>0.038± 0.008</td><td>15.30±0.04</td></tr>
<tr><td>Average</td><td>0.16± 0.04</td><td>0.65± 0.08</td><td>0.44± 0.04</td><td>2.49± 0.09</td><td>0.11± 0.03</td><td>0.57± 0.04</td><td>0.15± 0.02</td><td>0.81± 0.06</td><td>0.89± 0.07</td><td>0.18± 0.02</td><td>0.031± 0.009</td><td>13.13±0.03</td></tr>
</table>

Table S3: Concentration of all individual phenols (expressed in mg/mL of equivalents of gallic acid) found in all extracts and inks following the recipe Cordoba.

Table S3a) Extracts before heating

<table>
<tr><th>Recipe</th><th>|Gallic Acid Glucoside|</th><th>|Gallic Acid|</th><th>|Digalloyl glucose|</th><th>|Digalloyl glucose|</th><th>|Digalloyl glucose|</th><th>|Digallic Acid|</th><th>|Digallic Acid|</th><th>|Trigalloyl glucose|</th><th>|Trigalloyl glucose |</th><th>|Trigallic Acid|</th><th>|Tetragalloyl glucose|</th><th>|Tetragalloyl glucose|</th></tr>
<tr><td>Cordoba1</td><td>0.26± 0.01</td><td>5.00± 0.02</td><td>0.240± 0.004</td><td>0.26± 0.02</td><td>0.32± 0.02</td><td>0.05± 0.01</td><td>0.78± 0.05</td><td>0.77± 0.04</td><td>0.40± 0.07</td><td>0.0110± 0.0001</td><td>0.030± 0.001</td><td>0.0098± 0.0001</td></tr>
<tr><td>Cordoba2</td><td>0.354± 0.005</td><td>5.0± 0.1</td><td>0.27± 0.01</td><td>0.34± 0.01</td><td>0.36± 0.02</td><td>0.0282± 0.0006</td><td>0.52± 0.02</td><td>0.51± 0.02</td><td>0.28± 0.02</td><td>0.0104± 0.0008</td><td>0.031± 0.003</td><td>0.0084± 0.0003</td></tr>
<tr><td>Cordoba3</td><td>0.447± 0.003</td><td>5.349± 0.008</td><td>0.319± 0.008</td><td>0.39± 0.04</td><td>0.26± 0.02</td><td>0.0022± 0.0002</td><td>0.13± 0.01</td><td>0.094± 0.008</td><td>0.10± 0.01</td><td>0.0100± 0.0002</td><td>0.0270± 0.0007</td><td>0.0113± 0.0005</td></tr>
<tr><td>Cordoba4</td><td>0.362± 0.006</td><td>4.02± 0.03</td><td>0.26± 0.01</td><td>0.3± 0.1</td><td>0.09± 0.02</td><td>0.065± 0.006</td><td>0.284± 0.007</td><td>0.22± 0.02</td><td>0.37± 0.02</td><td>0.0106± 0.0002</td><td>0.0323± 0.0005</td><td>0.016± 0.002</td></tr>
<tr><td>Cordoba5</td><td>0.50± 0.02</td><td>5.33± 0.06</td><td>0.41± 0.04</td><td>0.46± 0.04</td><td>0.25± 0.01</td><td>0.032± 0.005</td><td>0.198± 0.0006</td><td>0.14± 0.02</td><td>0.25± 0.04</td><td>0.0127± 0.0004</td><td>0.026± 0.002</td><td>0.011± 0.001</td></tr>
<tr><td>Average</td><td>0.385± 0.008</td><td>4.94± 0.04</td><td>0.30± 0.02</td><td>0.36± 0.05</td><td>0.25± 0.02</td><td>0.035± 0.005</td><td>0.38± 0.02</td><td>0.35± 0.02</td><td>0.28± 0.03</td><td>0.0109± 0.0004</td><td>0.029± 0.001</td><td>0.0115± 0.0008</td></tr>
</table>

<table>
<tr><th>Recipe</th><th>|PGG|</th><th>|PGG|</th><th>|PGG|</th><th>|Hexagalloyl glucose|</th><th>|Hexagalloyl glucose|</th><th>|PGG|</th><th>|Hexagalloyl glucose|</th><th>|Hexagalloyl glucose|</th><th>|Heptagalloyl glucose |</th><th>|Heptagalloyl glucose |</th><th>Sum</th></tr>
<tr><td>Cordoba1</td><td>0.0086± 0.0001</td><td>0.0030± 0.0001</td><td>0.0149± 0.0002</td><td>0.010± 0.003</td><td>0.0035± 0.0001</td><td>0.0592± 0.0002</td><td>0.12± 0.01</td><td>0.0003± 0.0002</td><td>0.0181± 0.0005</td><td>0.0014± 0.0005</td><td>8.36±0.01</td></tr>
<tr><td>Cordoba2</td><td>0.0092± 0.0008</td><td>0.003± 0.001</td><td>0.009± 0.001</td><td>0.0113± 0.0005</td><td>0.0038± 0.0002</td><td>0.064± 0.002</td><td>0.129± 0.004</td><td><LOQ</td><td>0.0071± 0.0005</td><td>0.0018± 0.0003</td><td>7.95±0.01</td></tr>
<tr><td>Cordoba3</td><td>0.0101± 0.0001</td><td>0.00237± 0.00003</td><td>0.0091± 0.0009</td><td>0.0105± 0.0004</td><td>0.0040± 0.0003</td><td>0.068± 0.002</td><td>0.134± 0.005</td><td>0.00014± 0.00002</td><td>0.0082± 0.0004</td><td>0.0019± 0.0001</td><td>7.394±0.005</td></tr>
<tr><td>Cordoba4</td><td>0.0107± 0.0004</td><td>0.0021± 0.0001</td><td>0.017± 0.002</td><td>0.0094± 0.0001</td><td>0.008± 0.001</td><td>0.076± 0.003</td><td>0.130± 0.007</td><td><LOQ</td><td>0.020± 0.002</td><td>0.0022± 0.0004</td><td>6.36±0.01</td></tr>
<tr><td>Cordoba5</td><td>0.008± 0.002</td><td>0.0012± 0.0002</td><td>0.0098± 0.0003</td><td>0.0044± 0.0002</td><td>0.007± 0.001</td><td>0.10± 0.02</td><td>0.158± 0.006</td><td><LOQ</td><td>0.021± 0.001</td><td>0.0011± 0.0004</td><td>7.94±0.01</td></tr>
<tr><td>Average</td><td>0.0094± 0.0006</td><td>0.0023± 0.0003</td><td>0.0120± 0.0009</td><td>0.0091± 0.0008</td><td>0.0051± 0.0006</td><td>0.073± 0.006</td><td>0.134± 0.007</td><td>0.0002± 0.0001</td><td>0.0147± 0.0009</td><td>0.0017± 0.0003</td><td>7.60±0.01</td></tr>
</table>

Table S3b) Extracts after heating

<table>
<tr><th>Recipe</th><th>|Gallic Acid Glucoside|</th><th>|Gallic Acid|</th><th>|Digalloyl glucose|</th><th>|Digalloyl glucose|</th><th>|Digalloyl glucose|</th><th>|Digallic Acid|</th><th>|Digallic Acid|</th><th>|Trigalloyl glucose|</th><th>|Trigalloyl glucose |</th><th>|Trigallic Acid|</th><th>|Tetragalloyl glucose|</th><th>|Tetragalloyl glucose|</th></tr>
<tr><td>Cordoba1</td><td>0.243± 0.009</td><td>4.157± 0.001</td><td>0.22± 0.02</td><td>0.213± 0.008</td><td>0.22± 0.01</td><td>0.053± 0.006</td><td>0.49± 0.02</td><td>0.86± 0.02</td><td>0.43± 0.02</td><td>0.0176± 0.0004</td><td>0.01099± 0.00008</td><td>0.00274± 0.00001</td></tr>
<tr><td>Cordoba2</td><td>0.34± 0.02</td><td>4.55± 0.04</td><td>0.265± 0.006</td><td>0.28± 0.02</td><td>0.32± 0.02</td><td>0.032± 0.006</td><td>0.59± 0.02</td><td>0.63± 0.03</td><td>0.26± 0.03</td><td>0.011± 0.001</td><td>0.01046± 0.00007</td><td>0.00289± 0.00003</td></tr>
<tr><td>Cordoba3</td><td>0.387± 0.006</td><td>4.79± 0.03</td><td>0.348± 0.002</td><td>0.38± 0.02</td><td>0.28± 0.01</td><td>0.0362± 0.0001</td><td>0.26± 0.01</td><td>0.26± 0.02</td><td>0.140± 0.004</td><td>0.017± 0.001</td><td>0.0109± 0.0002</td><td>0.00296± 0.00009</td></tr>
<tr><td>Cordoba4</td><td>0.310± 0.005</td><td>4.85± 0.02</td><td>0.25± 0.01</td><td>0.28± 0.02</td><td>0.26± 0.03</td><td>0.055± 0.009</td><td>0.29± 0.03</td><td>0.31± 0.02</td><td>0.29± 0.01</td><td>0.012± 0.001</td><td>0.013± 0.001</td><td>0.00305± 0.00008</td></tr>
<tr><td>Cordoba5</td><td>0.345± 0.008</td><td>5.51± 0.05</td><td>0.484± 0.002</td><td>0.49± 0.03</td><td>0.344± 0.009</td><td>0.0338± 0.0008</td><td>0.30± 0.02</td><td>0.19± 0.01</td><td>0.17± 0.02</td><td>0.013± 0.001</td><td>0.01084± 0.00003</td><td>0.0041± 0.0001</td></tr>
<tr><td>Average</td><td>0.326± 0.009</td><td>4.77± 0.03</td><td>0.314± 0.008</td><td>0.33± 0.02</td><td>0.28± 0.02</td><td>0.042± 0.004</td><td>0.38± 0.02</td><td>0.45± 0.02</td><td>0.26± 0.02</td><td>0.0142± 0.0009</td><td>0.0113± 0.0003</td><td>0.0315± 0.00006</td></tr>
</table>

<table>
<tr><th>Recipe</th><th>|PGG|</th><th>|PGG|</th><th>|PGG|</th><th>|Hexagalloyl glucose|</th><th>|Hexagalloyl glucose|</th><th>|PGG|</th><th>|Hexagalloyl glucose|</th><th>|Hexagalloyl glucose|</th><th>|Heptagalloyl glucose |</th><th>|Heptagalloyl glucose |</th><th>Sum</th></tr>
<tr><td>Cordoba1</td><td>0.0035± 0.0002</td><td>0.0269± 0.0003</td><td>0.0512± 0.0004</td><td><LOQ</td><td>0.0058± 0.0002</td><td>0.103± 0.009</td><td>0.066± 0.002</td><td><LOQ</td><td>0.038± 0.005</td><td>0.0115± 0.0008</td><td>7.233±0.007</td></tr>
<tr><td>Cordoba2</td><td>0.0041± 0.0001</td><td>0.0233± 0.0006</td><td>0.0459± 0.0004</td><td><LOQ</td><td>0.0074± 0.0005</td><td>0.067± 0.002</td><td>0.073± 0.003</td><td><LOQ</td><td>0.0371± 0.0007</td><td>0.0079± 0.0004</td><td>7.564±0.009</td></tr>
<tr><td>Cordoba3</td><td>0.00393± 0.00003</td><td>0.02510± 0.00004</td><td>0.0409± 0.0002</td><td><LOQ</td><td>0.0069± 0.0002</td><td>0.079± 0.001</td><td>0.073± 0.003</td><td><LOQ</td><td>0.03430± 0.00001</td><td>0.00640± 0.00005</td><td>7.166±0.005</td></tr>
<tr><td>Cordoba4</td><td>0.0026± 0.0005</td><td>0.0397± 0.0006</td><td>0.039± 0.006</td><td><LOQ</td><td>0.0060± 0.0005</td><td>0.139± 0.004</td><td>0.065± 0.002</td><td><LOQ</td><td>0.038± 0.004</td><td>0.0115± 0.0004</td><td>7.268±0.008</td></tr>
<tr><td>Cordoba5</td><td>0.0043± 0.0003</td><td>0.023± 0.008</td><td>0.053± 0.001</td><td>0.00154± 0.00009</td><td>0.0087± 0.0002</td><td>0.070± 0.003</td><td>0.072± 0.003</td><td><LOQ</td><td>0.0315± 0.0002</td><td>0.0108± 0.0005</td><td>8.171±0.008</td></tr>
<tr><td>Average</td><td>0.0037± 0.0002</td><td>0.028± 0.002</td><td>0.046± 0.002</td><td>0.00154± 0.00009</td><td>0.0069± 0.0003</td><td>0.092± 0.004</td><td>0.070± 0.002</td><td>--</td><td>0.036± 0.002</td><td>0.0096± 0.0004</td><td>7.480±0.007</td></tr>
</table>

Table S3c) Inks without gum arabic

<table>
<tr><th>Recipe</th><th>|Gallic Acid Glucoside|</th><th>|Gallic Acid|</th><th>|Digalloyl glucose|</th><th>|Digalloyl glucose|</th><th>|Digalloyl glucose|</th><th>|Digallic Acid|</th><th>|Digallic Acid|</th><th>|Trigalloyl glucose|</th><th>|Trigalloyl glucose |</th><th>|Trigallic Acid|</th><th>|Tetragalloyl glucose|</th><th>|Tetragalloyl glucose|</th></tr>
<tr><td>Cordoba1</td><td>0.140±
0.009</td><td>2.36±
0.01</td><td>0.042±
0.001</td><td>0.030±
0.005</td><td>0.04±
0.02</td><td><LOQ</td><td>0.036±
0.001</td><td>0.058±
0.006</td><td>0.0089±
0.0005</td><td><LOQ</td><td><LOQ</td><td><LOQ</td></tr>
<tr><td>Cordoba2</td><td>0.136±
0.001</td><td>1.949±
0.007</td><td>0.008±
0.002</td><td>0.0065±
0.0009</td><td>0.010±
0.003</td><td><LOQ</td><td>0.039±
0.003</td><td>0.058±
0.005</td><td>0.0031±
0.0002</td><td>0.0002±
0.0001</td><td><LOQ</td><td><LOQ</td></tr>
<tr><td>Cordoba3</td><td>0.175±
0.003</td><td>2.24±
0.03</td><td>0.0258±
0.0005</td><td>0.0209±
0.0006</td><td>0.0259±
0.0005</td><td><LOQ</td><td>0.012±
0.003</td><td>0.021±
0.002</td><td><LOQ</td><td><LOQ</td><td><LOQ</td><td><LOQ</td></tr>
<tr><td>Cordoba4</td><td>0.1577±
0.0006</td><td>2.364±
0.007</td><td>0.0189±
0.0009</td><td>0.0154±
0.0004</td><td>0.0160±
0.0007</td><td><LOQ</td><td>0.0043±
0.0004</td><td>0.0229±
0.0002</td><td><LOQ</td><td>0.0004±
0.0001</td><td><LOQ</td><td><LOQ</td></tr>
<tr><td>Cordoba5</td><td>0.189±
0.001</td><td>2.24±
0.02</td><td>0.0189±
0.0009</td><td>0.0154±
0.0004</td><td>0.0160±
0.0007</td><td><LOQ</td><td>0.006±
0.003</td><td>0.0130±
0.0004</td><td><LOQ</td><td>0.002±
0.001</td><td><LOQ</td><td><LOQ</td></tr>
<tr><td>Average</td><td>0.159±
0.003</td><td>2.23±
0.01</td><td>0.023±
0.001</td><td>0.018±
0.001</td><td>0.022±
0.004</td><td>--</td><td>0.019±
0.002</td><td>0.035±
0.003</td><td>0.0060±
0.0003</td><td>0.0010±
0.0006</td><td>--</td><td>--</td></tr>
</table>

<table>
<tr><th>Recipe</th><th>|PGG|</th><th>|PGG|</th><th>|PGG|</th><th>|Hexagalloyl glucose|</th><th>|Hexagalloyl glucose</th><th>|PGG|</th><th>|Hexagalloyl glucose|</th><th>|Hexagalloyl glucose|</th><th>|Heptagalloyl glucose |</th><th>|Heptagalloyl glucose |</th><th>Sum</th></tr>
<tr><td>Cordoba1</td><td><LOQ</td><td><LOQ</td><td>0.0057±
0.0005</td><td><LOQ</td><td><LOQ</td><td>0.0105±
0.0005</td><td>0.019±
0.002</td><td><LOQ</td><td><LOQ</td><td><LOQ</td><td>2.745±0.005</td></tr>
<tr><td>Cordoba2</td><td><LOQ</td><td><LOQ</td><td>0.0016±
0.0006</td><td><LOQ</td><td><LOQ</td><td>0.0075±
0.0002</td><td>0.012±
0.002</td><td><LOQ</td><td><LOQ</td><td><LOQ</td><td>2.230±0.002</td></tr>
<tr><td>Cordoba3</td><td><LOQ</td><td><LOQ</td><td>0.0026±
0.0002</td><td><LOQ</td><td><LOQ</td><td>0.00393±
0.00002</td><td>0.0096±
0.0002</td><td><LOQ</td><td><LOQ</td><td><LOQ</td><td>2.538±0.004</td></tr>
<tr><td>Cordoba4</td><td><LOQ</td><td><LOQ</td><td>0.022±
0.004</td><td><LOQ</td><td><LOQ</td><td>0.006±
0.001</td><td>0.0121±
0.0006</td><td><LOQ</td><td><LOQ</td><td><LOQ</td><td>2.640±0.001</td></tr>
<tr><td>Cordoba5</td><td><LOQ</td><td><LOQ</td><td>0.0042±
0.0009</td><td><LOQ</td><td><LOQ</td><td>0.0001±
0.0007</td><td>0.0094±
0.0003</td><td><LOQ</td><td><LOQ</td><td><LOQ</td><td>2.509±0.003</td></tr>
<tr><td>Average</td><td>--</td><td>--</td><td>0.007±
0.001</td><td>--</td><td>--</td><td>0.001±
0.0005</td><td>0.012±
0.001</td><td>--</td><td>--</td><td>--</td><td>2.532±0.003</td></tr>
</table>

Table S3d) Inks with gum arabic

<table>
<tr><th>Recipe</th><th>|Gallic Acid Glucoside|</th><th>|Gallic Acid|</th><th>|Digalloyl glucose|</th><th>|Digalloyl glucose|</th><th>|Digalloyl glucose|</th><th>|Digallic Acid|</th><th>|Digallic Acid|</th><th>|Trigalloyl glucose|</th><th>|Trigalloyl glucose |</th><th>|Trigallic Acid|</th><th>|Tetragalloyl glucose|</th><th>|Tetragalloyl glucose|</th></tr>
<tr><td>Cordoba1</td><td>0.106± 0.004</td><td>1.76± 0.01</td><td>0.009± 0.001</td><td>0.00834± 0.00008</td><td>0.016± 0.002</td><td><LOQ</td><td>0.0097± 0.0003</td><td>0.095± 0.005</td><td>0.0076± 0.0004</td><td><LOQ</td><td><LOQ</td><td><LOQ</td></tr>
<tr><td>Cordoba2</td><td>0.129± 0.004</td><td>1.793± 0.007</td><td>0.014± 0.001</td><td>0.013± 0.004</td><td>0.015± 0.001</td><td><LOQ</td><td>0.010± 0.001</td><td>0.074± 0.004</td><td>0.0021± 0.0006</td><td><LOQ</td><td><LOQ</td><td><LOQ</td></tr>
<tr><td>Cordoba3</td><td>0.154± 0.005</td><td>1.67± 0.02</td><td>0.0128± 0.0007</td><td>0.011± 0.004</td><td>0.009± 0.002</td><td><LOQ</td><td>0.010± 0.002</td><td>0.024± 0.004</td><td></td><td><LOQ</td><td><LOQ</td><td><LOQ</td></tr>
<tr><td>Cordoba4</td><td>0.096± 0.006</td><td>1.66± 0.02</td><td>0.008± 0.001</td><td>0.001± 0.001</td><td>0.0072± 0.0004</td><td><LOQ</td><td>0.007± 0.002</td><td>0.021± 0.008</td><td>0.007± 0.004</td><td><LOQ</td><td><LOQ</td><td><LOQ</td></tr>
<tr><td>Cordoba5</td><td>0.185± 0.007</td><td>2.00± 0.02</td><td>0.024± 0.001</td><td>0.021± 0.001</td><td>0.016± 0.002</td><td><LOQ</td><td>0.010± 0.003</td><td>0.021± 0.004</td><td>0.0010± 0.0004</td><td><LOQ</td><td><LOQ</td><td><LOQ</td></tr>
<tr><td>Average</td><td>0.134± 0.005</td><td>1.78± 0.02</td><td>0.014± 0.001</td><td>0.011± 0.002</td><td>0.013± 0.002</td><td>--</td><td>0.009± 0.002</td><td>0.047± 0.005</td><td>0.004± 0.001</td><td>--</td><td>--</td><td>--</td></tr>
</table>

<table>
<tr><th>Recipe</th><th>|PGG|</th><th>|PGG|</th><th>|PGG|</th><th>|Hexagalloyl glucose|</th><th>|Hexagalloyl glucose|</th><th>|PGG|</th><th>|Hexagalloyl glucose|</th><th>|Hexagalloyl glucose|</th><th>|Heptagalloyl glucose |</th><th>|Heptagalloyl glucose |</th><th>Sum</th></tr>
<tr><td>Cordoba1</td><td><LOQ</td><td><LOQ</td><td>0.0098± 0.004</td><td><LOQ</td><td><LOQ</td><td>0.0141± 0.0007</td><td>0.032± 0.003</td><td><LOQ</td><td>0.0012± 0.0002</td><td><LOQ</td><td>2.072±0.003</td></tr>
<tr><td>Cordoba2</td><td><LOQ</td><td><LOQ</td><td>0.0037± 0.0003</td><td><LOQ</td><td><LOQ</td><td>0.010± 0.001</td><td>0.015± 0.002</td><td><LOQ</td><td><LOQ</td><td><LOQ</td><td>2.080±0.002</td></tr>
<tr><td>Cordoba3</td><td><LOQ</td><td><LOQ</td><td>0.0037± 0.0001</td><td><LOQ</td><td><LOQ</td><td>0.0074± 0.0003</td><td>0.0096± 0.0008</td><td><LOQ</td><td><LOQ</td><td><LOQ</td><td>1.915±0.003</td></tr>
<tr><td>Cordoba4</td><td><LOQ</td><td><LOQ</td><td>0.0049± 0.0004</td><td><LOQ</td><td><LOQ</td><td>0.0065± 0.0007</td><td>0.04± 0.02</td><td><LOQ</td><td>0.0003± 0.0001</td><td><LOQ</td><td>1.857±0.005</td></tr>
<tr><td>Cordoba5</td><td><LOQ</td><td><LOQ</td><td>0.020± 0.002</td><td><LOQ</td><td><LOQ</td><td>0.005± 0.001</td><td>0.006± 0.002</td><td><LOQ</td><td><LOQ</td><td><LOQ</td><td>2.311±0.004</td></tr>
<tr><td>Average</td><td>--</td><td>--</td><td>0.0084± 0.0006</td><td>--</td><td>--</td><td>0.0086± 0.0009</td><td>0.020± 0.005</td><td>--</td><td>0.0008± 0.0001</td><td>--</td><td>2.047±0.003</td></tr>
</table>

Table S4: Concentration of all individual phenols (expressed in mg/mL of equivalents of gallic acid) found in all extracts and inks following the recipe Guadalupe.

Table S4a) Extracts before heating

Recipe	|Gallic Acid Glucoside|	|Gallic Acid|	|Digalloyl glucose|	|Digalloyl glucose|	|Digalloyl glucose|	|Digallic Acid|	|Digallic Acid|	Digallic Acid|	|Trigalloyl glucose |	|Trigalloyl glucose |	|Trigalloyl glucose |	|Trigallic Acid|	|Tetragalloyl glucose|	|Tetragalloyl glucose|
Guadalupe1	0.85± 0.03	5.00± 0.2	0.26± 0.02	0.22± 0.02	0.15± 0.02	0.35± 0.01	0.59± 0.03	1.6± 0.1	0.37± 0.03	1.85± 0.07	0.8± 0.1	0.3± 0.1	4.0± 0.1	0.84± 0.01
Guadalupe2	0.71± 0.03	5.0± 0.1	0.23± 0.04	0.18± 0.01	0.115± 0.02	0.46± 0.0006	0.54± 0.06	1.4± 0.1	0.347± 0.001	1.65± 0.05	0.61± 0.08	0.38± 0.01	3.9± 0.1	0.80± 0.04
Guadalupe3	0.94± 0.04	5.349± 0.2	0.10± 0.04	0.09± 0.04	0.06± 0.02	0.36± 0.0002	0.52± 0.05	0.76± 0.07	0.28± 0.03	0.86± 0.09	0.30± 0.03	0.96± 0.08	5.363± 0.005	0.62± 0.02
Guadalupe4	0.95± 0.05	4.02± 0.3	0.31± 0.03	0.18± 0.1	0.17± 0.02	0.51± 0.006	0.59± 0.01	1.78± 0.01	0.33± 0.07	2.22± 0.08	0.99± 0.06	1.06± 0.07	3.9± 0.1	0.92± 0.03
Guadalupe5	1.33± 0.05	5.33± 0.2	0.87± 0.04	0.071± 0.04	0.048± 0.01	0.440± 0.005	0.19± 0.03	3.2± 0.2	0.16± 0.01	3.9± 0.1	1.3± 0.1	0.17± 0.02	4.156± 0.004	0.36± 0.04
Average	0.96± 0.04	4.94± 0.2	0.36± 0.04	0.15± 0.05	0.11± 0.02	0.43± 0.005	0.49± 0.04	1.76± 0.09	0.30± 0.03	2.09± 0.08	0.79± 0.08	0.58± 0.06	4.25± 0.07	0.71± 0.03

Recipe	|PGG|	|PGG|	|PGG|	|PGG|	|Hexagalloyl glucose|	|Hexagalloyl glucose|	|Hexagalloyl glucose|	|Hexagalloyl glucose|	|Hexagalloyl glucose|	|Heptagalloyl glucose |	|Heptagalloyl glucose |	|Diethyl gallate|	Sum
Guadalupe1	1.8± 0.1	1.73± 0.09	12.0± 0.2	0.09± 0.02	0.70± 0.05	1.74± 0.04	0.94± 0.04	3.0± 0.1	4.2± 0.1	0.61± 0.06	0.24± 0.06	0.36± 0.02	51.19±0.07
Guadalupe2	1.8± 0.1	1.7± 0.1	116± 0.1	0.11± 0.01	0.66± 0.03	1.87± 0.03	0.93± 0.03	2.9± 0.1	4.1± 0.1	0.6± 0.1	0.25± 0.02	0.34± 0.07	47.60±0.06
Guadalupe3	2.5± 0.1	1.89± 0.04	12.0± 0.3	0.119± 0.004	0.89± 0.01	1.50± 0.04	0.93± 0.06	2. 9± 0.1	4.00± 0.09	0.55± 0.03	0.239± 0.008	0.31± 0.04	45.17±0.06
Guadalupe4	1.73± 0.08	1.98± 0.08	13.0± 0.3	0.070± 0.001	0.54± 0.06	1.5± 0.1	0.9± 0.1	3.3± 0.1	4.8± 0.1	0.64± 0.07	0.25± 0.06	0.36± 0.04	54.78±0.08
Guadalupe5	1.75± 0.05	1.41± 0.07	13.1± 0.2	0.070± 0.002	0.77± 0.01	1.5± 0.1	0.43± 0.01	2.82± 0.07	3.7± 0.1	0.37± 0.05	0.23± 0.01	0.2279± 0.0008	51.79±0.06
Average	1.92± 0.09	1.74± 0.08	12.4± 0.2	0.092± 0.008	0.71± 0.03	1.60± 0.06	0.82± 0.06	3.07± 0.1	4.1± 0.1	0.56± 0.07	0.24± 0.03	0.32± 0.04	50.10±0.07

Table S4b) Extracts after heating

<table>
<tr><th>Recipe</th><th>|Gallic Acid Glucoside|</th><th>|Gallic Acid|</th><th>|Digalloyl glucose|</th><th>|Digalloyl glucose|</th><th>|Digalloyl glucose|</th><th>|Digallic Acid|</th><th>|Digallic Acid|</th><th>Digallic Acid|</th><th>|Trigalloyl glucose |</th><th>|Trigalloyl glucose |</th><th>|Trigalloyl glucose |</th><th>|Trigallic Acid|</th><th>|Tetragalloyl glucose|</th><th>|Tetragalloyl glucose|</th></tr>
<tr><td>Guadalupe1</td><td>0.69± 0.03</td><td>3.25± 0.02</td><td><LOQ</td><td><LOQ</td><td><LOQ</td><td>0.48± 0.05</td><td>0.80± 0.01</td><td>0.12± 0.02</td><td>0.310± 0.001</td><td>0.25± 0.01</td><td>0.10± 0.01</td><td>1.34± 0.02</td><td>7.5± 0.2</td><td>0.52± 0.02</td></tr>
<tr><td>Guadalupe2</td><td>0.58± 0.02</td><td>3.52± 0.07</td><td><LOQ</td><td><LOQ</td><td><LOQ</td><td>0.448± 0.004</td><td>0.65± 0.01</td><td>0.23± 0.01</td><td>0.28± 0.01</td><td>0.38± 0.02</td><td>0.059± 0.002</td><td>1.00± 0.02</td><td>5.98± 0.02</td><td>0.43± 0.07</td></tr>
<tr><td>Guadalupe3</td><td>2.21± 0.03</td><td>2.936± 0.005</td><td>0.32± 0.04</td><td>2.3± 0.1</td><td>2.69± 0.06</td><td>0.27± 0.03</td><td>0.43± 0.02</td><td>0.191± 0.003</td><td>0.42± 0.02</td><td>1.0± 0.1</td><td>0.0184± 0.0003</td><td>0.20± 0.01</td><td>6.9± 0.01</td><td>0.445± 0.2</td></tr>
<tr><td>Guadalupe4</td><td>0.94± 0.04</td><td>5.603± 0.002</td><td>0.25± 0.03</td><td>2.42± 0.05</td><td>2.10± 0.07</td><td>0.52± 0.02</td><td>0.62± 0.06</td><td>0.106± 0.005</td><td>0.31± 0.04</td><td>1.31± 0.09</td><td>0.41± 0.07</td><td>0.293± 0.007</td><td>5.4± 0.007</td><td>0.78± 0.1</td></tr>
<tr><td>Guadalupe5</td><td>1.03± 0.04</td><td>4.492± 0.003</td><td>0.42± 0.02</td><td>2.42± 0.05</td><td>2.59± 0.05</td><td>0.33± 0.02</td><td>0.61± 0.01</td><td>0.229± 0.005</td><td>0.209± 0.004</td><td>0.28± 0.03</td><td>1.10± 0.03</td><td>0.19± 0.07</td><td>6.2± 0.07</td><td>0.51± 0.2</td></tr>
<tr><td>Average</td><td>1.09± 0.03</td><td>3.96± 0.02</td><td>0.33± 0.03</td><td>2.39± 0.08</td><td>2.46± 0.06</td><td>0.41± 0.03</td><td>0.62± 0.02</td><td>0.175± 0.009</td><td>0.31± 0.02</td><td>0.64± 0.05</td><td>0.34± 0.02</td><td>0.61± 0.03</td><td>6.4± 0.03</td><td>0.54± 0.2</td></tr>
</table>

<table>
<tr><th>Recipe</th><th>|PGG|</th><th>|PGG|</th><th>|PGG|</th><th>|PGG|</th><th>|Hexagalloyl glucose|</th><th>|Hexagalloyl glucose|</th><th>|Hexagalloyl glucose|</th><th>|Hexagalloyl glucose|</th><th>|Hexagalloyl glucose|</th><th>|Heptagalloyl glucose |</th><th>|Heptagalloyl glucose |</th><th>|Diethyl gallate|</th><th>Sum</th></tr>
<tr><td>Guadalupe1</td><td>4.699± 0.006</td><td>2.39± 0.07</td><td>14.8± 0.1</td><td>0.20± 0.02</td><td>1.43± 0.04</td><td>2.13± 0.07</td><td>1.17± 0.09</td><td>3.427± 0.004</td><td>4.62± 0.05</td><td>0.36± 0.03</td><td>0.29± 0.01</td><td>0.41± 0.01</td><td>51.57±0.04</td></tr>
<tr><td>Guadalupe2</td><td>3.7± 0.1</td><td>1.98± 0.08</td><td>12.4± 0.3</td><td>0.16± 0.04</td><td>1.10± 0.05</td><td>2.0± 0.1</td><td>0.98± 0.06</td><td>3.01± 0.06</td><td>4.10± 0.05</td><td>0.38± 0.03</td><td>0.274± 0.008</td><td>0.27± 0.03</td><td>44.16±0.05</td></tr>
<tr><td>Guadalupe3</td><td>4.10± 0.06</td><td>2.03± 0.03</td><td>12.1± 0.2</td><td>0.185± 0.001</td><td><LOQ</td><td>1.46± 0.04</td><td>1.47± 0.03</td><td>2.82± 0.06</td><td>3.06± 0.09</td><td>0.31± 0.01</td><td>0.300± 0.008</td><td>0.185± 0.008</td><td>48.57±0.05</td></tr>
<tr><td>Guadalupe4</td><td>3.26± 0.03</td><td>2.27± 0.03</td><td>14.1± 0.2</td><td>0.19± 0.01</td><td>1.70± 0.05</td><td>1.1± 0.1</td><td>3.37± 0.05</td><td>4.8± 0.1</td><td>3.21± 0.06</td><td>0.30± 0.01</td><td>0.321± 0.008</td><td>0.161± 0.005</td><td>55.39±0.05</td></tr>
<tr><td>Guadalupe5</td><td>3.57± 0.06</td><td>2.21± 0.05</td><td>13.75± 0.09</td><td>0.206± 0.001</td><td>1.50± 0.04</td><td>1.83± 0.03</td><td>2.28± 0.02</td><td>3.36± 0.09</td><td>3.50± 0.03</td><td>0.291± 0.0005</td><td>0.283± 0.003</td><td>0.252± 0.006</td><td>53.90±0.04</td></tr>
<tr><td>Average</td><td>3.87± 0.06</td><td>2.17± 0.05</td><td>13.4± 0.2</td><td>0.19± 0.01</td><td>1.43± 0.05</td><td>1.70± 0.07</td><td>1.85± 0.05</td><td>3.47± 0.07</td><td>3.70± 0.06</td><td>0.33± 0.02</td><td>0.293± 0.008</td><td>0.25± 0.01</td><td>50.82±0.05</td></tr>
</table>

Table S4c) Inks without gum arabic

Recipe	|Gallic Acid Glucoside|	|Gallic Acid|	|Digalloyl glucose|	|Digalloyl glucose|	|Digalloyl glucose|	|Digallic Acid|	|Digallic Acid|	Digallic Acid|	|Trigalloyl glucose |	|Trigalloyl glucose |	|Trigalloyl glucose |	|Trigallic Acid|	|Tetragalloyl glucose|	|Tetragalloyl glucose|
**Guadalupe1**	0.48± 0.01	1.853± 0.006	<LOQ	<LOQ	<LOQ	0.115± 0.001	0.31± 0.02	0.005± 0.001	0.041± 0.008	0.005± 0.002	0.007± 0.001	0.06± 0.01	0.52± 0.09	0.07± 0.01
**Guadalupe2**	0.41± 0.03	1.73± 0.03	<LOQ	<LOQ	<LOQ	0.09± 0.01	0.273± 0.009	0.003± 0.002	0.039± 0.009	0.006± 0.002	0.007± 0.001	0.07± 0.01	0.83± 0.09	0.07± 0.01
**Guadalupe3**	0.402± 0.004	1.61± 0.02	<LOQ	<LOQ	<LOQ	0.0812± 0.0009	0.25± 0.01	<LOQ	0.030± 0.003	0.0056± 0.0007	0.0063± 0.0007	0.060± 0.002	0.6± 0. 1	0.040± 0.002
**Guadalupe4**	0.43± 0.01	1.56± 0.02	<LOQ	<LOQ	<LOQ	0.09± 0.02	0.27± 0.02	0.002± 0.002	0.033± 0.006	0.007± 0.004	0.0104± 0.0006	0.056± 0.006	0.8± 0.1	0.08± 0.01
**Guadalupe5**	0.45± 0.01	1.593± 0.004	<LOQ	<LOQ	<LOQ	0.136± 0.008	0.30± 0.02	0.0121± 0.0009	0.047± 0.007	0.0077± 0.0008	0.011± 0.002	0.048± 0.006	0.6± 0.1	0.052± 0.004
**Average**	0.43± 0.01	1.67± 0.02	--	--	--	0.101± 0.008	0.28± 0.01	0.006± 0.001	0.038± 0.007	0.006± 0.002	0.008± 0.001	0.058± 0.007	0.7± 0.1	0.063± 0.008

Recipe	|PGG|	|PGG|	|PGG|	|PGG|	|Hexagalloyl glucose|	|Hexagalloyl glucose|	|Hexagalloyl glucose|	|Hexagalloyl glucose|	|Hexagalloyl glucose|	|Heptagalloyl glucose |	|Heptagalloyl glucose |	|Diethyl gallate|	**Sum**
**Guadalupe1**	0.38± 0.03	0.12± 0.09	1.05± 0.07	0.0073± 0.0008	0.09± 0.07	0.14± 0.01	0.08± 0.06	0.20± 0.03	0.28± 0.02	0.012± 0.001	0.04± 0.03	0.020± 0.002	5.88±0.03
**Guadalupe2**	0.47± 0.03	0.2± 0.1	1.33± 0.07	0.0115± 0.0009	0.12± 0.07	0.19± 0.01	0.10± 0.07	0.27± 0.05	0.50± 0.01	0.019± 0.001	0.06± 0.04	0.019± 0.001	6.79±0.03
**Guadalupe3**	0.27± 0.09	0.11± 0.02	0.93± 0.06	0.004± 0.001	0.07± 0.02	0.09± 0.03	0.055± 0.005	0.15± 0.05	0.22± 0.06	0.006± 0.001	0.03± 0.01	0.009± 0.005	5.05±0.02
**Guadalupe4**	0.4± 0.1	0.16± 0.07	1.15± 0.05	0.0052± 0.0004	0.09± 0.04	0.11± 0.04	0.08± 0.05	0.19± 0.07	0.38± 0.03	0.010± 0.006	0.04± 0.02	0.011± 0.006	5.94±0.03
**Guadalupe5**	0.39± 0.04	0.18± 0.01	1.25± 0.04	0.005± 0.001	0.07± 0.05	0.09± 0.06	0.07± 0.05	0.17± 0.09	0.035± 0.001	0.007± 0.007	0.03± 0.03	0.010± 0.001	5.58±0.02
**Average**	0.37± 0.06	0.15± 0.06	1.14± 0.06	0.007± 0.001	0.09± 0.05	0.12± 0.03	0.08± 0.05	0.20± 0.06	0.29± 0.02	0.011± 0.003	0.04± 0.03	0.014± 0.003	5.85±0.03

Table S4d) Inks with gum arabic

<table>
<tr><th>Recipe</th><th>|Gallic Acid Glucoside|</th><th>|Gallic Acid|</th><th>|Digalloyl glucose|</th><th>|Digalloyl glucose|</th><th>|Digalloyl glucose|</th><th>|Digallic Acid|</th><th>|Digallic Acid|</th><th>Digallic Acid|</th><th>|Trigalloyl glucose |</th><th>|Trigalloyl glucose |</th><th>|Trigalloyl glucose |</th><th>|Trigallic Acid|</th><th>|Tetragalloyl glucose|</th><th>|Tetragalloyl glucose|</th></tr>
<tr><td>Guadalupe1</td><td>0.37± 0.05</td><td>1.4± 0.1</td><td><LOQ</td><td><LOQ</td><td><LOQ</td><td>0.065± 0.003</td><td>0.27± 0.05</td><td><LOQ</td><td>0.04± 0.03</td><td>0.015± 0.003</td><td>0.019± 0.002</td><td>0.09± 0.04</td><td>1.14± 0.02</td><td>0.13± 0.04</td></tr>
<tr><td>Guadalupe2</td><td>0.36± 0.03</td><td>1.46± 0.05</td><td><LOQ</td><td><LOQ</td><td><LOQ</td><td>0.08± 0.01</td><td>0.24± 0.03</td><td>0.0008± 0.0001</td><td>0.046± 0.008</td><td>0.010± 0.003</td><td>0.014± 0.005</td><td>0.08± 0.03</td><td>0.85± 0.04</td><td>0.09± 0.02</td></tr>
<tr><td>Guadalupe3</td><td>0.31± 0.02</td><td>1.57± 0.03</td><td><LOQ</td><td><LOQ</td><td><LOQ</td><td>0.061± 0.004</td><td>0.199± 0.005</td><td><LOQ</td><td>0.034± 0.008</td><td>0.02± 0.01</td><td>0.02± 0.01</td><td>0.12± 0.03</td><td>1.02± 0.04</td><td>0.12± 0.08</td></tr>
<tr><td>Guadalupe4</td><td>0.39± 0.02</td><td>1.40± 0.08</td><td><LOQ</td><td><LOQ</td><td><LOQ</td><td>0.086± 0.006</td><td>0.261± 0.004</td><td>0.002± 0.001</td><td>0.040± 0.004</td><td>0.018± 0.005</td><td>0.018± 0.008</td><td>0.11± 0.06</td><td>0.88± 0.05</td><td>0.11± 0.03</td></tr>
<tr><td>Guadalupe5</td><td>0.38± 0.02</td><td>1.33± 0.05</td><td><LOQ</td><td><LOQ</td><td><LOQ</td><td>0.07± 0.01</td><td>0.27± 0.01</td><td><LOQ</td><td>0.040± 0.005</td><td>0.021± 0.006</td><td>0.019± 0.003</td><td>0.09± 0.07</td><td>0.89± 0.03</td><td>0.14± 0.05</td></tr>
<tr><td>Average</td><td>0.36± 0.03</td><td>1.44± 0.06</td><td>--</td><td>--</td><td>--</td><td>0.073± 0.007</td><td>0.25± 0.02</td><td>0.001± 0.001</td><td>0.04± 0.01</td><td>0.018± 0.006</td><td>0.018± 0.006</td><td>0.10± 0.05</td><td>0.96± 0.04</td><td>0.12± 0.04</td></tr>
</table>

<table>
<tr><th>Recipe</th><th>|PGG|</th><th>|PGG|</th><th>|PGG|</th><th>|PGG|</th><th>|Hexagalloyl glucose|</th><th>|Hexagalloyl glucose|</th><th>|Hexagalloyl glucose|</th><th>|Hexagalloyl glucose|</th><th>|Hexagalloyl glucose|</th><th>|Heptagalloyl glucose |</th><th>|Heptagalloyl glucose |</th><th>|Diethyl gallate|</th><th>Sum</th></tr>
<tr><td>Guadalupe1</td><td>0.53± 0.02</td><td>0.22± 0.03</td><td>2.1± 0.1</td><td>0.009± 0.006</td><td>0.11± 0.02</td><td>0.21± 0.09</td><td>0.09± 0.06</td><td>0.29± 0.04</td><td>0.566± 0.05</td><td>0.01± 0.002</td><td>0.042± 0.009</td><td>0.02± 0.01</td><td>7.83±0.04</td></tr>
<tr><td>Guadalupe2</td><td>0.374± 0.001</td><td>0.18± 0.05</td><td>1.90± 0.05</td><td>0.007± 0.002</td><td>0.07± 0.04</td><td>0.16± 0.02</td><td>0.07± 0.01</td><td>0.28± 0.09</td><td>0.424± 0.03</td><td>0.011± 0.003</td><td>0.045± 0.002</td><td>0.014± 0.001</td><td>6.76±0.02</td></tr>
<tr><td>Guadalupe3</td><td>0.55± 0.03</td><td>0.21± 0.05</td><td>2.11± 0.08</td><td>0.008± 0.001</td><td>0.12± 0.03</td><td>0.16± 0.03</td><td>0.10± 0.01</td><td>0.27± 0.02</td><td>0.488± 0.02</td><td>0.02± 0.002</td><td>0.05± 0.05</td><td>0.02± 0.01</td><td>7.58±0.03</td></tr>
<tr><td>Guadalupe4</td><td>0.37± 0.02</td><td>0.19± 0.02</td><td>1.7± 0.3</td><td>0.003± 0.001</td><td>0.07± 0.03</td><td>0.12± 0.01</td><td>0.07± 0.02</td><td>0.27± 0.08</td><td>0.432± 0.03</td><td>0.06± 0.004</td><td>0.038± 0.006</td><td>0.012± 0.002</td><td>6.65±0.03</td></tr>
<tr><td>Guadalupe5</td><td>0.58± 0.03</td><td>0.24± 0.09</td><td>2.26± 0.09</td><td>0.008± 0.008</td><td>0.13± 0.01</td><td>0.18± 0.08</td><td>0.11± 0.08</td><td>0.32± 0.08</td><td>0.560± 0.01</td><td>0.01± 0.01</td><td>0.06± 0.03</td><td>0.02± 0.01</td><td>7.73±0.04</td></tr>
<tr><td>Average</td><td>0.48± 0.02</td><td>0.21± 0.05</td><td>2.0± 0.1</td><td>0.007± 0.004</td><td>0.10± 0.03</td><td>0.17± 0.05</td><td>0.09± 0.04</td><td>0.29± 0.06</td><td>0.494± 0.03</td><td>0.02± 0.005</td><td>0.05± 0.02</td><td>0.017± 0.009</td><td>7.31±0.03</td></tr>
</table>

Table S5: Concentration of all individual phenols (expressed in mg/mL of equivalents of gallic acid) found in all extracts and inks following the recipe Madrid.

Table S5a) Extracts

Recipe	|Gallic Acid Glucoside|	|Gallic Acid|	|Digallic Acid|	|Digallic Acid|	|Trigalloyl glucose |	|Trigalloyl glucose |	|Trigallic Acid|	|Tetragalloyl glucose|	|Tetragalloyl glucose|	|PGG|	|PGG|
**Madrid1**	0.183± 0.006	1.017± 0.008	0.13± 0.01	0.27± 0.02	0.22± 0.02	0.082± 0.003	0.39± 0.05	3.33± 0.06	0.13± 0.02	1.60± 0.03	0.92± 0.04
**Madrid2**	0.171± 0.007	0.97± 0.01	0.134± 0.009	0.23± 0.03	0.209± 0.009	0.088± 0.005	0.35± 0.02	3.24± 0.07	0.156± 0.004	1.67± 0.05	0.88± 0.02
**Madrid3**	0.20± 0.02	1.02± 0.01	0.14± 0.02	0.30± 0.02	0.217± 0.008	0.071± 0.006	0.436± 0.007	3.13± 0.03	0.15± 0.01	1.65± 0.01	0.77± 0.02
**Madrid4**	0.212± 0.003	0.95± 0.08	0.16± 0.01	0.304± 0.004	0.22± 0.01	0.073± 0.009	0.43± 0.02	3.16± 0.04	0.171± 0.009	1.63± 0.06	0.79± 0.04
**Madrid5**	0.205± 0.008	1.030± 0.009	0.15± 0.01	0.299± 0.007	0.203± 0.007	0.08± 0.01	0.44± 0.02	3.28± 0.03	0.159± 0.008	1.70± 0.01	0.86± 0.03
**Average**	0.194± 0.009	1.00± 0.02	0.14± 0.01	0.28± 0.02	0.21± 0.01	0.079± 0.006	0.41± 0.02	3.23± 0.05	0.15± 0.01	1.65± 0.03	0.85± 0.03

Recipe	|PGG|	|PGG|	|Hexagalloyl glucose|	|Hexagalloyl glucose|	|Hexagalloyl glucose|	|Hexagalloyl glucose|	|Hexagalloyl glucose|	|Heptagalloyl glucose|	|Heptagalloyl glucose|	|Diethyl gallate|	**Sum**
**Madrid1**	4.47± 0.06	0.07± 0.02	0.44± 0.02	0.72± 0.02	0.35± 0.04	1.20± 0.03	2.05± 0.03	0.28± 0.01	0.133± 0.002	0.16± 0.01	18.15±0.02
**Madrid2**	4.33± 0.09	0.06± 0.01	0.41± 0.07	0.76± 0.06	0.39± 0.02	1.23± 0.06	2.06± 0.06	0.20± 0.03	0.10± 0.05	0.16± 0.02	17.81±0.03
**Madrid3**	3.77± 0.05	0.07± 0.01	0.49± 0.01	0.75± 0.01	0.403± 0.004	1.06± 0.05	1.853± 0.004	0.29± 0.03	0.126± 0.002	0.15± 0.02	17.04±0.02
**Madrid4**	4.12± 0.01	0.08± 0.01	0.486± 0.002	0.71± 0.02	0.42± 0.02	1.19± 0.02	1.96± 0.03	0.28± 0.04	0.127± 0.008	0.158± 0.008	17.65±0.02
**Madrid5**	4.27± 0.06	0.10± 0.01	0.52± 0.01	0.80± 0.05	0.43± 0.03	1.21± 0.05	2.01± 0.05	0.35± 0.07	0.139± 0.004	0.16± 0.01	18.40±0.02
**Average**	4.19± 0.06	0.08± 0.01	0.47± 0.02	0.75± 0.03	0.40± 0.02	1.18± 0.04	1.99± 0.03	0.28± 0.04	0.13± 0.01	0.16± 0.01	17.81±0.02

Table S5b) Inks without gum arabic

Recipe	|Gallic Acid Glucoside|	|Gallic Acid|	|Digallic Acid|	|Digallic Acid|	|Trigalloyl glucose |	|Trigalloyl glucose |	|Trigallic Acid|	|Tetragalloyl glucose|	|Tetragalloyl glucose|	|PGG|	|PGG|
**Madrid1**	0.32± 0.01	0.94± 0.03	0.11± 0.01	0.23± 0.04	0.148± 0.009	0.067± 0.004	0.246± 0.005	1.95± 0.07	0.096± 0.001	0.77± 0.03	0.459± 0.009
**Madrid2**	0.34± 0.01	0.88± 0.01	0.108± 0.009	0.23± 0.01	0.149± 0.001	0.073± 0.002	0.20± 0.02	1.88± 0.09	0.08± 0.01	0.71± 0.03	0.46± 0.02
**Madrid3**	0.45± 0.03	0.98± 0.03	0.095± 0.001	0.26± 0.02	0.12± 0.03	0.04± 0.01	0.22± 0.02	1.53± 0.03	0.08± 0.02	0.72± 0.05	0.29± 0.05
**Madrid4**	0.42± 0.02	0.92± 0.02	0.11± 0.01	0.22± 0.02	0.11± 0.01	0.044± 0.001	0.12± 0.04	1.93± 0.02	0.096± 0.006	0.46± 0.06	0.280± 0.006
**Madrid5**	0.423± 0.004	0.97± 0.02	0.111± 0.007	0.24± 0.02	0.137± 0.007	0.058± 0.008	0.25± 0.02	1.88± 0.06	0.104± 0.003	0.77± 0.07	0.36± 0.07
**Average**	0.39± 0.02	0.94± 0.02	0.106± 0.009	0.24± 0.02	0.13± 0.01	0.056± 0.006	0.21± 0.02	1.83± 0.05	0.09± 0.01	0.69± 0.05	0.37± 0.03

Recipe	|PGG|	|PGG|	|Hexagalloyl glucose|	|Hexagalloyl glucose|	|Hexagalloyl glucose|	|Hexagalloyl glucose|	|Hexagalloyl glucose|	|Heptagalloyl glucose|	|Heptagalloyl glucose|	|Diethyl gallate|	**Sum**
**Madrid1**	3.0± 0.1	0.0031± 0.0005	0.17± 0.01	0.46± 0.01	0.22± 0.02	0.8± 0.1	0.69± 0.02	0.15± 0.03	0.075± 0.008	0.085± 0.008	10.96±0.03
**Madrid2**	2.79± 0.08	<LOQ	0.16± 0.02	0.38± 0.04	0.18± 0.04	0.77± 0.07	1.28± 0.05	0.13± 0.01	0.07± 0.01	0.088± 0.003	10.95±0.03
**Madrid3**	1.94± 0.03	<LOQ	0.16± 0.04	0.29± 0.04	0.159± 0.008	0.51± 0.08	0.87± 0.06	0.11± 0.01	0.044± 0.008	0.061± 0.006	8.92±0.03
**Madrid4**	2.2± 0.1	<LOQ	0.190± 0.009	0.310± 0.001	0.164± 0.002	0.61± 0.01	0.926± 0.008	0.128± 0.007	0.061± 0.001	0.073± 0.001	9.34±0.02
**Madrid5**	2.30± 0.05	<LOQ	0.17± 0.05	0.31± 0.07	0.20± 0.05	0.70± 0.04	1.18± 0.04	0.14± 0.04	0.05± 0.02	0.081± 0.007	10.45±0.03
**Average**	2.44± 0.08	0.0031± 0.0005	0.17± 0.03	0.35± 0.03	0.18± 0.03	0.68± 0.06	0.99± 0.04	0.13± 0.02	0.060± 0.009	0.078± 0.005	10.12±0.03

Table S5c) Inks with gum arabic

Recipe	|Gallic Acid Glucoside|	|Gallic Acid|	|Digallic Acid|	|Digallic Acid|	|Trigalloyl glucose |	|Trigalloyl glucose |	|Trigallic Acid|	|Tetragalloyl glucose|	|Tetragalloyl glucose|	|PGG|	|PGG|
**Madrid1**	0.53± 0.03	0.83± 0.04	0.13± 0.07	0.210± 0.009	0.090± 0.008	0.046± 0.006	0.19± 0.02	1.61± 0.01	0.111± 0.005	0.61± 0.05	0.33± 0.03
**Madrid2**	0.50± 0.03	0.92± 0.01	0.083± 0.006	0.228± 0.003	0.10± 0.01	0.04± 0.01	0.25± 0.02	1.57± 0.01	0.098± 0.004	0.62± 0.03	0.352± 0.007
**Madrid3**	0.48± 0.02	0.88± 0.04	0.07± 0.007	0.21± 0.01	0.094± 0.006	0.030± 0.003	0.20± 0.03	1.21± 0.02	0.101± 0.009	0.52± 0.01	0.26± 0.01
**Madrid4**	0.63± 0.02	1.03± 0.02	0.080± 0.02	0.23± 0.04	0.09± 0.03	0.031± 0.007	0.30± 0.02	1.70± 0.04	0.13± 0.02	0.72± 0.03	0.33± 0.05
**Madrid5**	0.57± 0.02	0.83± 0.08	0.080± 0.005	0.21± 0.01	0.07± 0.02	0.028± 0.005	0.24± 0.03	1.41± 0.02	0.10± 0.02	0.63± 0.02	0.28± 0.03
**Average**	0.54± 0.02	0.90± 0.04	0.09± 0.02	0.22± 0.02	0.09± 0.01	0.036± 0.006	0.23± 0.02	1.50± 0.02	0.11± 0.01	0.62± 0.03	0.31± 0.03

Recipe	|PGG|	|PGG|	|Hexagalloyl glucose|	|Hexagalloyl glucose|	|Hexagalloyl glucose|	|Hexagalloyl glucose|	|Hexagalloyl glucose|	|Heptagalloyl glucose|	|Heptagalloyl glucose|	|Diethyl gallate|	**Sum**
**Madrid1**	2.14± 0.06	<LOQ	0.15± 0.02	0.26± 0.03	0.17± 0.02	0.54± 0.06	0.99± 0.02	0.10± 0.03	0.050± 0.008	0.068± 0.004	9.13±0.03
**Madrid2**	2.13± 0.07	<LOQ	0.15± 0.01	0.26± 0.03	0.16± 0.01	0.57± 0.01	0.90± 0.01	0.115± 0.004	0.04± 0.01	0.06± 0.02	9.14±0.02
**Madrid3**	1.71± 0.05	<LOQ	0.125± 0.005	0.21± 0.03	0.134± 0.008	0.43± 0.03	0.73± 0.04	0.08± 0.02	0.030± 0.009	0.054± 0.004	7.55±0.02
**Madrid4**	2.25± 0.07	<LOQ	0.18± 0.02	0.283± 0.003	0.19± 0.01	0.57± 0.02	1.00± 0.06	0.10± 0.04	0.06± 0.01	0.0560± 0.0004	9.94±0.03
**Madrid5**	1.97± 0.08	<LOQ	0.15± 0.02	0.266± 0.008	0.15± 0.01	0.47± 0.04	0.84± 0.03	0.11± 0.02	0.041± 0.007	0.062± 0.003	8.57±0.02
**Average**	2.04± 0.07	--	0.15± 0.01	0.26± 0.02	0.16± 0.01	0.51± 0.03	0.89± 0.03	0.10± 0.02	0.04± 0.01	0.059± 0.007	8.86±0.02

Transcription of the selected Iberian writing ink recipes – from (Díaz Hidalgo et al., 2018)

- Arcebispado de **Braga**, cx.31:

"Recepta de tinta Toma de gallhas I onça e quebranta as meudas e lança as a ferver em hua libra d'auga terçada de vinagre branco e fervam tamto que mingue as II partes e lança lhe meia onça de goma e toma de azeche IIIIo onças muudo e peneirado e lança lho dentro e mexe com huum paao e folge assy hua noite e huum dia e será muito booa" (Melo, 2016).

3.12g of galls were ground and added to 33mL of millipore water and 16.6mL of white vinegar and boiled, until it is reduced to two parts of the volume. After 1.56g of gum arabic were added, under stirring until it is completely dissolved, after which 12.5g of previously sifted $FeSO_4$ were added to the solution. The solution was stirred with a branch from a fig tree and left to settle one night and one day.

- **Montpellier** fol.233v:

"Para tinta. Toma una libra de acije y 1 libra y media de agallas y 1 libra de goma. Rompe las agallas y ponlas en remojo en tres cuartillos de agua durante 3 días. Después cuece y reduce casi 3 partes de agua y si hierve cuélala. Una vez colada mete dentro una onza de goma y revuelve hasta que se ponga líquida toda la goma. Después en dicha agua fría mete 1 onza del dicho acije y remueve y déjalo estar así durante un día. Después cuélala y métela en ampollas, etcetera" (Rouchon, Belhadj, Duranton, Gimat, & Massiani, 2016).

1.43g of galls were crushed and added to 50mL of millipore water and left to extract at room temperature for 3 days. After this time, the solution was heated and reduced to 1⁄4 and then filtered. 0.95g of ground arabic gum were added, under stirring until dissolution, and, after cooling, 0.95g of $FeSO_4$ were added. It was left to settle for one day and then filtered.

- Protocolos **Córdoba** fol.58v:

"Para faser tinta buena toma vna olla de vn asunbre e echalde tres quartillos de agua e echalde dos onças de agallas bien quebradas e contía de vna honça de cáscaras de granadas agras sy las oviere o sy non sean de granadas duses e estén en remojo contía de ocho días. E después coseldas sobre fuego de carbón muy manso quanto escomiençe a feruir e non más, e después dexaldas esfriar e desque fuere bien fría sacáredes las agallas coladas e espremidas e echaldas fuera. E tomad dos honças de buen asiche e moleldo e echaldo en vna haltanna e cobrildo en agua e esté fasta que sea desfecho e echaldo en el agua de las agallas e meçello muy bien e dexaldo asentar vn día, e después colalda con vna vedija de lana que cayga en otra olla. E en lo colado echáredes vna honça de buena goma e dende a dos días será fecha" (Kroustallis, 2007).

1.918g of well crushed galls and 0.959g of pomegranate peel were added to 50mL of millipore water and left to extract for 8 days at room temperature. After this period, the solution was slowly heated until boiling point and after cooling it was filtrated. 1.91g of $FeSO_4$ were placed in a glass cup and covered in water and dissolved, and this solution was later added to the extraction. The solution was left to settle for one day and then filtrated. After, gum arabic was added and left to settle for two days.

- Libro de los Oficios de **Guadalupe** fol.201r-v:

"Para un açumbre de tinta son menester seys honças de agallas e quatro honças de201 v// azige, e dos ho[nças de goma, e dos de caparroso.] As de saber que echamos t[anta agua como tin]ta queremos sacar, salvo que anidimos [a un a]cumbre de agua un quartillo de vino [blanco] puro, bueno para el cozer, e por esta man[era] podéis acresçentar o amenguar. E a[se de] fazer en esta manera, echada el agua e vino en una olla limpia, todo junto, has de echar en una escudilla las agallas bien partidas, e en otra el azige, e en otra la goma, e del agua que está en la olla con el vino, echa sobre el azige fasta que se cubra, e en la goma eso mismo cada uno aparte, e en el agua que quedare, echa las agallas. E así lo has de tener çinco o seis días, meçiéndolo muchas vezes con un palo de higuera cada uno por sý. Después desto, echa las agallas con su agua en una olla e ponlas sobre el fuego, el qual a de ser manso, e cuézelas tanto fasta que le pueda dezir dos vezes el salmo de miserere mey, e has de ser avisado que no se salga por çima al ferner. E después tira la olla del fuego e déxala un poquito, e échala en otra olla202 r// [colándola, e luego, antes de que se en]fríe, échale [dentro la goma e méçela] bien porque desha[ga, e después] de otro poco, antes que se acabe de [enf]riar, échale el azije, mirando que no eches [la tierra,] más que se quede en el suelo de la escudilla, [e después] que todo esté bien frío, has de tener la capa[rros]a molyda e échasela dentro, e dende a [un] rato tórnalo a colar, e échalo en las vasijas en que ha de estar" (Vitorino, Melo, Carlyle, & Otero, 2016).

5.6g of crushed galls, 3.75g of $FeSO_4$ and 1.87g of gum arabic were placed in three separate glass cups. 37.5mL of millipore water and white wine (12.5mL of wine) were distributed by the cups in the following manner: enough solution to cover the $FeSO_4$ and the gum arabic and the rest for the galls. The solutions were left to settle for 6 days, stirring every day with a dried branch from a fig tree. After this time, the galls solution was heated with low temperature for 10 minutes (60ºC) and filtered. After a while, and without letting it be too cold, the $FeSO_4$ and gum arabic solutions were added, always under stirring. After being at room temperature, 1.87g of $CuSO_4$ were added and the solution was filtrated.

- Ms 9226 de **Madrid**, fol.192r-v:

"Para haber un açumbre de tinta echa açumbre y medio de vino blanco doncel 6 onzas de agallas quebrantada[s] y dexalas estar 9 dias meneándolas mucho. [Luego] al cabo de ellos cuela este vino en otra olla y echa en el 6 onças de caparrosa y otras 6 de goma. Luego echa Açucar piedra y piedra alumbre y añir de cada [¿esta?] media onça y tenla

al sol dos o tres días mene[an]dolo bien, o a fuego manso. Y cuando este echa guardala en vaso limpio en lugar fresco" (Barreiros, 1838).

2.21g of crushed galls were added to 40mL of white wine and left for 9 days, at room temperature. After this time, the solution was filtered. 2.21g of $FeSO_4$ and 2.21g of gum arabic were added to the solution, as well as 0.18g of brown sugar, 0.18g of indigo and 0.18g of alumen, and the solution was slightly heated at 60ºC.

LITERATURE CITED

Barreiros, J. F. (1838). *Memória sobre os pesos e medidas de Portugal,Espanha, Inglaterra e França: que se empregão nos trabalhos do corpo de engenheiros e da arma de artilheria.* Lisboa: Typog. da Acad. Real das Sciencias.

Díaz Hidalgo, R. J., Córdoba, R., Nabais, P., Silva, V., Melo, M. J., Pina, F., . . . Freitas, V. (2018). New insights into iron-gall inks through the use of historically accurate reconstructions. *Heritage Science, 6*(1), 63. https://doi.org/10.1186/s40494-018-0228-8.

Kroustallis, S. (2007). El oficio de Pergaminería y el Reglamento del Scriptorium del Monasterio de Ntra. Sra. de Guadalupe. In M. S. Brinquis, Cabanes, M.L., editors. (Ed.), *Libro de los Oficios del Monasterio de Nuestra Señora de Guadalupe.* (pp. 246-247). Badajoz:: Secretaría General Técnica del Ministerio de Cultura, Subdirección General de Publicaciones, Información y Docu- mentación, Junta de Extremadura, Consejería de Cultura y Turismo y Monasterio de Guadalupe.

Melo, M. J., Castro, R.,editors. (2016). The "Book on How to Make Colours", o livro de como se fazem as cores das tintas todas. Medieval Colours for Practitioners. Retrieved from: https://www.dcr.fct.unl.pt/LivComoFazemCores Accessed 2020.

Rouchon, V., Belhadj, O., Duranton, M., Gimat, A., & Massiani, P. (2016). Application of Arrhenius law to DP and zero-span tensile strength measurements taken on iron gall ink impregnated papers: relevance of artificial ageing protocols. *Applied Physics A, 122*(8), 773. https://doi.org/10.1007/s00339-016-0307-1.

Vitorino, T., Melo, M. J., Carlyle, L., & Otero, V. (2016). New insights into brazilwood lake pigments manufacture through the use of historically accurate reconstructions. *Studies in Conservation, 61*(5), 255-273. https://doi.org/10.1179/2047058415Y.0000000006.
